# Search for dark matter produced in association with bottom or top quarks in $\sqrt{s}=13$ TeV *pp* collisions with the ATLAS detector

**DOI:** 10.1140/epjc/s10052-017-5486-1

**Published:** 2018-01-11

**Authors:** M. Aaboud, G. Aad, B. Abbott, O. Abdinov, B. Abeloos, S. H. Abidi, O. S. AbouZeid, N. L. Abraham, H. Abramowicz, H. Abreu, R. Abreu, Y. Abulaiti, B. S. Acharya, S. Adachi, L. Adamczyk, J. Adelman, M. Adersberger, T. Adye, A. A. Affolder, Y. Afik, T. Agatonovic-Jovin, C. Agheorghiesei, J. A. Aguilar-Saavedra, S. P. Ahlen, F. Ahmadov, G. Aielli, S. Akatsuka, H. Akerstedt, T. P. A. Åkesson, E. Akilli, A. V. Akimov, G. L. Alberghi, J. Albert, P. Albicocco, M. J. Alconada Verzini, S. C. Alderweireldt, M. Aleksa, I. N. Aleksandrov, C. Alexa, G. Alexander, T. Alexopoulos, M. Alhroob, B. Ali, M. Aliev, G. Alimonti, J. Alison, S. P. Alkire, B. M. M. Allbrooke, B. W. Allen, P. P. Allport, A. Aloisio, A. Alonso, F. Alonso, C. Alpigiani, A. A. Alshehri, M. I. Alstaty, B. Alvarez Gonzalez, D. Álvarez Piqueras, M. G. Alviggi, B. T. Amadio, Y. Amaral Coutinho, C. Amelung, D. Amidei, S. P. Amor Dos Santos, S. Amoroso, C. Anastopoulos, L. S. Ancu, N. Andari, T. Andeen, C. F. Anders, J. K. Anders, K. J. Anderson, A. Andreazza, V. Andrei, S. Angelidakis, I. Angelozzi, A. Angerami, A. V. Anisenkov, N. Anjos, A. Annovi, C. Antel, M. Antonelli, A. Antonov, D. J. Antrim, F. Anulli, M. Aoki, L. Aperio Bella, G. Arabidze, Y. Arai, J. P. Araque, V. Araujo Ferraz, A. T. H. Arce, R. E. Ardell, F. A. Arduh, J.-F. Arguin, S. Argyropoulos, M. Arik, A. J. Armbruster, L. J. Armitage, O. Arnaez, H. Arnold, M. Arratia, O. Arslan, A. Artamonov, G. Artoni, S. Artz, S. Asai, N. Asbah, A. Ashkenazi, L. Asquith, K. Assamagan, R. Astalos, M. Atkinson, N. B. Atlay, K. Augsten, G. Avolio, B. Axen, M. K. Ayoub, G. Azuelos, A. E. Baas, M. J. Baca, H. Bachacou, K. Bachas, M. Backes, P. Bagnaia, M. Bahmani, H. Bahrasemani, J. T. Baines, M. Bajic, O. K. Baker, P. J. Bakker, E. M. Baldin, P. Balek, F. Balli, W. K. Balunas, E. Banas, A. Bandyopadhyay, Sw. Banerjee, A. A. E. Bannoura, L. Barak, E. L. Barberio, D. Barberis, M. Barbero, T. Barillari, M.-S. Barisits, J. T. Barkeloo, T. Barklow, N. Barlow, S. L. Barnes, B. M. Barnett, R. M. Barnett, Z. Barnovska-Blenessy, A. Baroncelli, G. Barone, A. J. Barr, L. Barranco Navarro, F. Barreiro, J. Barreiro Guimarães da Costa, R. Bartoldus, A. E. Barton, P. Bartos, A. Basalaev, A. Bassalat, R. L. Bates, S. J. Batista, J. R. Batley, M. Battaglia, M. Bauce, F. Bauer, H. S. Bawa, J. B. Beacham, M. D. Beattie, T. Beau, P. H. Beauchemin, P. Bechtle, H. P. Beck, H. C. Beck, K. Becker, M. Becker, C. Becot, A. J. Beddall, A. Beddall, V. A. Bednyakov, M. Bedognetti, C. P. Bee, T. A. Beermann, M. Begalli, M. Begel, J. K. Behr, A. S. Bell, G. Bella, L. Bellagamba, A. Bellerive, M. Bellomo, K. Belotskiy, O. Beltramello, N. L. Belyaev, O. Benary, D. Benchekroun, M. Bender, N. Benekos, Y. Benhammou, E. Benhar Noccioli, J. Benitez, D. P. Benjamin, M. Benoit, J. R. Bensinger, S. Bentvelsen, L. Beresford, M. Beretta, D. Berge, E. Bergeaas Kuutmann, N. Berger, L. J. Bergsten, J. Beringer, S. Berlendis, N. R. Bernard, G. Bernardi, C. Bernius, F. U. Bernlochner, T. Berry, P. Berta, C. Bertella, G. Bertoli, I. A. Bertram, C. Bertsche, G. J. Besjes, O. Bessidskaia Bylund, M. Bessner, N. Besson, A. Bethani, S. Bethke, A. Betti, A. J. Bevan, J. Beyer, R. M. Bianchi, O. Biebel, D. Biedermann, R. Bielski, K. Bierwagen, N. V. Biesuz, M. Biglietti, T. R. V. Billoud, H. Bilokon, M. Bindi, A. Bingul, C. Bini, S. Biondi, T. Bisanz, C. Bittrich, D. M. Bjergaard, J. E. Black, K. M. Black, R. E. Blair, T. Blazek, I. Bloch, C. Blocker, A. Blue, U. Blumenschein, S. Blunier, G. J. Bobbink, V. S. Bobrovnikov, S. S. Bocchetta, A. Bocci, C. Bock, M. Boehler, D. Boerner, D. Bogavac, A. G. Bogdanchikov, C. Bohm, V. Boisvert, P. Bokan, T. Bold, A. S. Boldyrev, A. E. Bolz, M. Bomben, M. Bona, M. Boonekamp, A. Borisov, G. Borissov, J. Bortfeldt, D. Bortoletto, V. Bortolotto, D. Boscherini, M. Bosman, J. D. Bossio Sola, J. Boudreau, E. V. Bouhova-Thacker, D. Boumediene, C. Bourdarios, S. K. Boutle, A. Boveia, J. Boyd, I. R. Boyko, A. J. Bozson, J. Bracinik, A. Brandt, G. Brandt, O. Brandt, F. Braren, U. Bratzler, B. Brau, J. E. Brau, W. D. Breaden Madden, K. Brendlinger, A. J. Brennan, L. Brenner, R. Brenner, S. Bressler, D. L. Briglin, T. M. Bristow, D. Britton, D. Britzger, F. M. Brochu, I. Brock, R. Brock, G. Brooijmans, T. Brooks, W. K. Brooks, J. Brosamer, E. Brost, J. H Broughton, P. A. Bruckman de Renstrom, D. Bruncko, A. Bruni, G. Bruni, L. S. Bruni, S. Bruno, B. H. Brunt, M. Bruschi, N. Bruscino, P. Bryant, L. Bryngemark, T. Buanes, Q. Buat, P. Buchholz, A. G. Buckley, I. A. Budagov, F. Buehrer, M. K. Bugge, O. Bulekov, D. Bullock, T. J. Burch, S. Burdin, C. D. Burgard, A. M. Burger, B. Burghgrave, K. Burka, S. Burke, I. Burmeister, J. T. P. Burr, D. Büscher, V. Büscher, P. Bussey, J. M. Butler, C. M. Buttar, J. M. Butterworth, P. Butti, W. Buttinger, A. Buzatu, A. R. Buzykaev, C.-Q. Changqiao, S. Cabrera Urbán, D. Caforio, H. Cai, V. M. Cairo, O. Cakir, N. Calace, P. Calafiura, A. Calandri, G. Calderini, P. Calfayan, G. Callea, L. P. Caloba, S. Calvente Lopez, D. Calvet, S. Calvet, T. P. Calvet, R. Camacho Toro, S. Camarda, P. Camarri, D. Cameron, R. Caminal Armadans, C. Camincher, S. Campana, M. Campanelli, A. Camplani, A. Campoverde, V. Canale, M. Cano Bret, J. Cantero, T. Cao, M. D. M. Capeans Garrido, I. Caprini, M. Caprini, M. Capua, R. M. Carbone, R. Cardarelli, F. Cardillo, I. Carli, T. Carli, G. Carlino, B. T. Carlson, L. Carminati, R. M. D. Carney, S. Caron, E. Carquin, S. Carrá, G. D. Carrillo-Montoya, D. Casadei, M. P. Casado, A. F. Casha, M. Casolino, D. W. Casper, R. Castelijn, V. Castillo Gimenez, N. F. Castro, A. Catinaccio, J. R. Catmore, A. Cattai, J. Caudron, V. Cavaliere, E. Cavallaro, D. Cavalli, M. Cavalli-Sforza, V. Cavasinni, E. Celebi, F. Ceradini, L. Cerda Alberich, A. S. Cerqueira, A. Cerri, L. Cerrito, F. Cerutti, A. Cervelli, S. A. Cetin, A. Chafaq, D. Chakraborty, S. K. Chan, W. S. Chan, Y. L. Chan, P. Chang, J. D. Chapman, D. G. Charlton, C. C. Chau, C. A. Chavez Barajas, S. Che, S. Cheatham, A. Chegwidden, S. Chekanov, S. V. Chekulaev, G. A. Chelkov, M. A. Chelstowska, C. Chen, C. Chen, H. Chen, J. Chen, S. Chen, S. Chen, X. Chen, Y. Chen, H. C. Cheng, H. J. Cheng, A. Cheplakov, E. Cheremushkina, R. Cherkaoui El Moursli, E. Cheu, K. Cheung, L. Chevalier, V. Chiarella, G. Chiarelli, G. Chiodini, A. S. Chisholm, A. Chitan, Y. H. Chiu, M. V. Chizhov, K. Choi, A. R. Chomont, S. Chouridou, Y. S. Chow, V. Christodoulou, M. C. Chu, J. Chudoba, A. J. Chuinard, J. J. Chwastowski, L. Chytka, A. K. Ciftci, D. Cinca, V. Cindro, I. A. Cioara, A. Ciocio, F. Cirotto, Z. H. Citron, M. Citterio, M. Ciubancan, A. Clark, B. L. Clark, M. R. Clark, P. J. Clark, R. N. Clarke, C. Clement, Y. Coadou, M. Cobal, A. Coccaro, J. Cochran, L. Colasurdo, B. Cole, A. P. Colijn, J. Collot, T. Colombo, P. Conde Muiño, E. Coniavitis, S. H. Connell, I. A. Connelly, S. Constantinescu, G. Conti, F. Conventi, M. Cooke, A. M. Cooper-Sarkar, F. Cormier, K. J. R. Cormier, M. Corradi, F. Corriveau, A. Cortes-Gonzalez, G. Costa, M. J. Costa, D. Costanzo, G. Cottin, G. Cowan, B. E. Cox, K. Cranmer, S. J. Crawley, R. A. Creager, G. Cree, S. Crépé-Renaudin, F. Crescioli, W. A. Cribbs, M. Cristinziani, V. Croft, G. Crosetti, A. Cueto, T. Cuhadar Donszelmann, A. R. Cukierman, J. Cummings, M. Curatolo, J. Cúth, S. Czekierda, P. Czodrowski, G. D’amen, S. D’Auria, L. D’eramo, M. D’Onofrio, M. J. Da Cunha Sargedas De Sousa, C. Da Via, W. Dabrowski, T. Dado, T. Dai, O. Dale, F. Dallaire, C. Dallapiccola, M. Dam, J. R. Dandoy, M. F. Daneri, N. P. Dang, A. C. Daniells, N. S. Dann, M. Danninger, M. Dano Hoffmann, V. Dao, G. Darbo, S. Darmora, J. Dassoulas, A. Dattagupta, T. Daubney, W. Davey, C. David, T. Davidek, D. R. Davis, P. Davison, E. Dawe, I. Dawson, K. De, R. de Asmundis, A. De Benedetti, S. De Castro, S. De Cecco, N. De Groot, P. de Jong, H. De la Torre, F. De Lorenzi, A. De Maria, D. De Pedis, A. De Salvo, U. De Sanctis, A. De Santo, K. De Vasconcelos Corga, J. B. De Vivie De Regie, R. Debbe, C. Debenedetti, D. V. Dedovich, N. Dehghanian, I. Deigaard, M. Del Gaudio, J. Del Peso, D. Delgove, F. Deliot, C. M. Delitzsch, A. Dell’Acqua, L. Dell’Asta, M. Dell’Orso, M. Della Pietra, D. della Volpe, M. Delmastro, C. Delporte, P. A. Delsart, D. A. DeMarco, S. Demers, M. Demichev, A. Demilly, S. P. Denisov, D. Denysiuk, D. Derendarz, J. E. Derkaoui, F. Derue, P. Dervan, K. Desch, C. Deterre, K. Dette, M. R. Devesa, P. O. Deviveiros, A. Dewhurst, S. Dhaliwal, F. A. Di Bello, A. Di Ciaccio, L. Di Ciaccio, W. K. Di Clemente, C. Di Donato, A. Di Girolamo, B. Di Girolamo, B. Di Micco, R. Di Nardo, K. F. Di Petrillo, A. Di Simone, R. Di Sipio, D. Di Valentino, C. Diaconu, M. Diamond, F. A. Dias, M. A. Diaz, J. Dickinson, E. B. Diehl, J. Dietrich, S. Díez Cornell, A. Dimitrievska, J. Dingfelder, P. Dita, S. Dita, F. Dittus, F. Djama, T. Djobava, J. I. Djuvsland, M. A. B. do Vale, D. Dobos, M. Dobre, D. Dodsworth, C. Doglioni, J. Dolejsi, Z. Dolezal, M. Donadelli, S. Donati, P. Dondero, J. Donini, J. Dopke, A. Doria, M. T. Dova, A. T. Doyle, E. Drechsler, M. Dris, Y. Du, J. Duarte-Campderros, F. Dubinin, A. Dubreuil, E. Duchovni, G. Duckeck, A. Ducourthial, O. A. Ducu, D. Duda, A. Dudarev, A. Chr. Dudder, E. M. Duffield, L. Duflot, M. Dührssen, C. Dulsen, M. Dumancic, A. E. Dumitriu, A. K. Duncan, M. Dunford, A. Duperrin, H. Duran Yildiz, M. Düren, A. Durglishvili, D. Duschinger, B. Dutta, D. Duvnjak, M. Dyndal, B. S. Dziedzic, C. Eckardt, K. M. Ecker, R. C. Edgar, T. Eifert, G. Eigen, K. Einsweiler, T. Ekelof, M. El Kacimi, R. El Kosseifi, V. Ellajosyula, M. Ellert, S. Elles, F. Ellinghaus, A. A. Elliot, N. Ellis, J. Elmsheuser, M. Elsing, D. Emeliyanov, Y. Enari, J. S. Ennis, M. B. Epland, J. Erdmann, A. Ereditato, M. Ernst, S. Errede, M. Escalier, C. Escobar, B. Esposito, O. Estrada Pastor, A. I. Etienvre, E. Etzion, H. Evans, A. Ezhilov, M. Ezzi, F. Fabbri, L. Fabbri, V. Fabiani, G. Facini, R. M. Fakhrutdinov, S. Falciano, R. J. Falla, J. Faltova, Y. Fang, M. Fanti, A. Farbin, A. Farilla, C. Farina, E. M. Farina, T. Farooque, S. Farrell, S. M. Farrington, P. Farthouat, F. Fassi, P. Fassnacht, D. Fassouliotis, M. Faucci Giannelli, A. Favareto, W. J. Fawcett, L. Fayard, O. L. Fedin, W. Fedorko, S. Feigl, L. Feligioni, C. Feng, E. J. Feng, M. J. Fenton, A. B. Fenyuk, L. Feremenga, P. Fernandez Martinez, J. Ferrando, A. Ferrari, P. Ferrari, R. Ferrari, D. E. Ferreira de Lima, A. Ferrer, D. Ferrere, C. Ferretti, F. Fiedler, A. Filipčič, M. Filipuzzi, F. Filthaut, M. Fincke-Keeler, K. D. Finelli, M. C. N. Fiolhais, L. Fiorini, A. Fischer, C. Fischer, J. Fischer, W. C. Fisher, N. Flaschel, I. Fleck, P. Fleischmann, R. R. M. Fletcher, T. Flick, B. M. Flierl, L. R. Flores Castillo, M. J. Flowerdew, G. T. Forcolin, A. Formica, F. A. Förster, A. Forti, A. G. Foster, D. Fournier, H. Fox, S. Fracchia, P. Francavilla, M. Franchini, S. Franchino, D. Francis, L. Franconi, M. Franklin, M. Frate, M. Fraternali, D. Freeborn, S. M. Fressard-Batraneanu, B. Freund, D. Froidevaux, J. A. Frost, C. Fukunaga, T. Fusayasu, J. Fuster, O. Gabizon, A. Gabrielli, A. Gabrielli, G. P. Gach, S. Gadatsch, S. Gadomski, G. Gagliardi, L. G. Gagnon, C. Galea, B. Galhardo, E. J. Gallas, B. J. Gallop, P. Gallus, G. Galster, K. K. Gan, S. Ganguly, Y. Gao, Y. S. Gao, F. M. Garay Walls, C. García, J. E. García Navarro, J. A. García Pascual, M. Garcia-Sciveres, R. W. Gardner, N. Garelli, V. Garonne, A. Gascon Bravo, K. Gasnikova, C. Gatti, A. Gaudiello, G. Gaudio, I. L. Gavrilenko, C. Gay, G. Gaycken, E. N. Gazis, C. N. P. Gee, J. Geisen, M. Geisen, M. P. Geisler, K. Gellerstedt, C. Gemme, M. H. Genest, C. Geng, S. Gentile, C. Gentsos, S. George, D. Gerbaudo, G. Geßner, S. Ghasemi, M. Ghneimat, B. Giacobbe, S. Giagu, N. Giangiacomi, P. Giannetti, S. M. Gibson, M. Gignac, M. Gilchriese, D. Gillberg, G. Gilles, D. M. Gingrich, M. P. Giordani, F. M. Giorgi, P. F. Giraud, P. Giromini, G. Giugliarelli, D. Giugni, F. Giuli, C. Giuliani, M. Giulini, B. K. Gjelsten, S. Gkaitatzis, I. Gkialas, E. L. Gkougkousis, P. Gkountoumis, L. K. Gladilin, C. Glasman, J. Glatzer, P. C. F. Glaysher, A. Glazov, M. Goblirsch-Kolb, J. Godlewski, S. Goldfarb, T. Golling, D. Golubkov, A. Gomes, R. Gonçalo, R. Goncalves Gama, J. Goncalves Pinto Firmino Da Costa, G. Gonella, L. Gonella, A. Gongadze, F. Gonnella, J. L. Gonski, S. González de la Hoz, S. Gonzalez-Sevilla, L. Goossens, P. A. Gorbounov, H. A. Gordon, B. Gorini, E. Gorini, A. Gorišek, A. T. Goshaw, C. Gössling, M. I. Gostkin, C. A. Gottardo, C. R. Goudet, D. Goujdami, A. G. Goussiou, N. Govender, E. Gozani, I. Grabowska-Bold, P. O. J. Gradin, J. Gramling, E. Gramstad, S. Grancagnolo, V. Gratchev, P. M. Gravila, C. Gray, H. M. Gray, Z. D. Greenwood, C. Grefe, K. Gregersen, I. M. Gregor, P. Grenier, K. Grevtsov, J. Griffiths, A. A. Grillo, K. Grimm, S. Grinstein, Ph. Gris, J.-F. Grivaz, S. Groh, E. Gross, J. Grosse-Knetter, G. C. Grossi, Z. J. Grout, A. Grummer, L. Guan, W. Guan, J. Guenther, F. Guescini, D. Guest, O. Gueta, B. Gui, E. Guido, T. Guillemin, S. Guindon, U. Gul, C. Gumpert, J. Guo, W. Guo, Y. Guo, R. Gupta, S. Gurbuz, G. Gustavino, B. J. Gutelman, P. Gutierrez, N. G. Gutierrez Ortiz, C. Gutschow, C. Guyot, M. P. Guzik, C. Gwenlan, C. B. Gwilliam, A. Haas, C. Haber, H. K. Hadavand, N. Haddad, A. Hadef, S. Hageböck, M. Hagihara, H. Hakobyan, M. Haleem, J. Haley, G. Halladjian, G. D. Hallewell, K. Hamacher, P. Hamal, K. Hamano, A. Hamilton, G. N. Hamity, P. G. Hamnett, L. Han, S. Han, K. Hanagaki, K. Hanawa, M. Hance, D. M. Handl, B. Haney, P. Hanke, J. B. Hansen, J. D. Hansen, M. C. Hansen, P. H. Hansen, K. Hara, A. S. Hard, T. Harenberg, F. Hariri, S. Harkusha, P. F. Harrison, N. M. Hartmann, Y. Hasegawa, A. Hasib, S. Hassani, S. Haug, R. Hauser, L. Hauswald, L. B. Havener, M. Havranek, C. M. Hawkes, R. J. Hawkings, D. Hayakawa, D. Hayden, C. P. Hays, J. M. Hays, H. S. Hayward, S. J. Haywood, S. J. Head, T. Heck, V. Hedberg, L. Heelan, S. Heer, K. K. Heidegger, S. Heim, T. Heim, B. Heinemann, J. J. Heinrich, L. Heinrich, C. Heinz, J. Hejbal, L. Helary, A. Held, S. Hellman, C. Helsens, R. C. W. Henderson, Y. Heng, S. Henkelmann, A. M. Henriques Correia, S. Henrot-Versille, G. H. Herbert, H. Herde, V. Herget, Y. Hernández Jiménez, H. Herr, G. Herten, R. Hertenberger, L. Hervas, T. C. Herwig, G. G. Hesketh, N. P. Hessey, J. W. Hetherly, S. Higashino, E. Higón-Rodriguez, K. Hildebrand, E. Hill, J. C. Hill, K. H. Hiller, S. J. Hillier, M. Hils, I. Hinchliffe, M. Hirose, D. Hirschbuehl, B. Hiti, O. Hladik, D. R. Hlaluku, X. Hoad, J. Hobbs, N. Hod, M. C. Hodgkinson, P. Hodgson, A. Hoecker, M. R. Hoeferkamp, F. Hoenig, D. Hohn, T. R. Holmes, M. Holzbock, M. Homann, S. Honda, T. Honda, T. M. Hong, B. H. Hooberman, W. H. Hopkins, Y. Horii, A. J. Horton, J.-Y. Hostachy, A. Hostiuc, S. Hou, A. Hoummada, J. Howarth, J. Hoya, M. Hrabovsky, J. Hrdinka, I. Hristova, J. Hrivnac, T. Hryn’ova, A. Hrynevich, P. J. Hsu, S.-C. Hsu, Q. Hu, S. Hu, Y. Huang, Z. Hubacek, F. Hubaut, F. Huegging, T. B. Huffman, E. W. Hughes, M. Huhtinen, R. F. H. Hunter, P. Huo, N. Huseynov, J. Huston, J. Huth, R. Hyneman, G. Iacobucci, G. Iakovidis, I. Ibragimov, L. Iconomidou-Fayard, Z. Idrissi, P. Iengo, O. Igonkina, T. Iizawa, Y. Ikegami, M. Ikeno, Y. Ilchenko, D. Iliadis, N. Ilic, F. Iltzsche, G. Introzzi, P. Ioannou, M. Iodice, K. Iordanidou, V. Ippolito, M. F. Isacson, N. Ishijima, M. Ishino, M. Ishitsuka, C. Issever, S. Istin, F. Ito, J. M. Iturbe Ponce, R. Iuppa, H. Iwasaki, J. M. Izen, V. Izzo, S. Jabbar, P. Jackson, R. M. Jacobs, V. Jain, K. B. Jakobi, K. Jakobs, S. Jakobsen, T. Jakoubek, D. O. Jamin, D. K. Jana, R. Jansky, J. Janssen, M. Janus, P. A. Janus, G. Jarlskog, N. Javadov, T. Javůrek, M. Javurkova, F. Jeanneau, L. Jeanty, J. Jejelava, A. Jelinskas, P. Jenni, C. Jeske, S. Jézéquel, H. Ji, J. Jia, H. Jiang, Y. Jiang, Z. Jiang, S. Jiggins, J. Jimenez Pena, S. Jin, A. Jinaru, O. Jinnouchi, H. Jivan, P. Johansson, K. A. Johns, C. A. Johnson, W. J. Johnson, K. Jon-And, R. W. L. Jones, S. D. Jones, S. Jones, T. J. Jones, J. Jongmanns, P. M. Jorge, J. Jovicevic, X. Ju, A. Juste Rozas, M. K. Köhler, A. Kaczmarska, M. Kado, H. Kagan, M. Kagan, S. J. Kahn, T. Kaji, E. Kajomovitz, C. W. Kalderon, A. Kaluza, S. Kama, A. Kamenshchikov, N. Kanaya, L. Kanjir, V. A. Kantserov, J. Kanzaki, B. Kaplan, L. S. Kaplan, D. Kar, K. Karakostas, N. Karastathis, M. J. Kareem, E. Karentzos, S. N. Karpov, Z. M. Karpova, V. Kartvelishvili, A. N. Karyukhin, K. Kasahara, L. Kashif, R. D. Kass, A. Kastanas, Y. Kataoka, C. Kato, A. Katre, J. Katzy, K. Kawade, K. Kawagoe, T. Kawamoto, G. Kawamura, E. F. Kay, V. F. Kazanin, R. Keeler, R. Kehoe, J. S. Keller, E. Kellermann, J. J. Kempster, J Kendrick, H. Keoshkerian, O. Kepka, B. P. Kerševan, S. Kersten, R. A. Keyes, M. Khader, F. Khalil-zada, A. Khanov, A. G. Kharlamov, T. Kharlamova, A. Khodinov, T. J. Khoo, V. Khovanskiy, E. Khramov, J. Khubua, S. Kido, C. R. Kilby, H. Y. Kim, S. H. Kim, Y. K. Kim, N. Kimura, O. M. Kind, B. T. King, D. Kirchmeier, J. Kirk, A. E. Kiryunin, T. Kishimoto, D. Kisielewska, V. Kitali, O. Kivernyk, E. Kladiva, T. Klapdor-Kleingrothaus, M. H. Klein, M. Klein, U. Klein, K. Kleinknecht, P. Klimek, A. Klimentov, R. Klingenberg, T. Klingl, T. Klioutchnikova, F. F. Klitzner, E.-E. Kluge, P. Kluit, S. Kluth, E. Kneringer, E. B. F. G. Knoops, A. Knue, A. Kobayashi, D. Kobayashi, T. Kobayashi, M. Kobel, M. Kocian, P. Kodys, T. Koffas, E. Koffeman, N. M. Köhler, T. Koi, M. Kolb, I. Koletsou, T. Kondo, N. Kondrashova, K. Köneke, A. C. König, T. Kono, R. Konoplich, N. Konstantinidis, B. Konya, R. Kopeliansky, S. Koperny, A. K. Kopp, K. Korcyl, K. Kordas, A. Korn, A. A. Korol, I. Korolkov, E. V. Korolkova, O. Kortner, S. Kortner, T. Kosek, V. V. Kostyukhin, A. Kotwal, A. Koulouris, A. Kourkoumeli-Charalampidi, C. Kourkoumelis, E. Kourlitis, V. Kouskoura, A. B. Kowalewska, R. Kowalewski, T. Z. Kowalski, C. Kozakai, W. Kozanecki, A. S. Kozhin, V. A. Kramarenko, G. Kramberger, D. Krasnopevtsev, M. W. Krasny, A. Krasznahorkay, D. Krauss, J. A. Kremer, J. Kretzschmar, K. Kreutzfeldt, P. Krieger, K. Krizka, K. Kroeninger, H. Kroha, J. Kroll, J. Kroll, J. Kroseberg, J. Krstic, U. Kruchonak, H. Krüger, N. Krumnack, M. C. Kruse, T. Kubota, H. Kucuk, S. Kuday, J. T. Kuechler, S. Kuehn, A. Kugel, F. Kuger, T. Kuhl, V. Kukhtin, R. Kukla, Y. Kulchitsky, S. Kuleshov, Y. P. Kulinich, M. Kuna, T. Kunigo, A. Kupco, T. Kupfer, O. Kuprash, H. Kurashige, L. L. Kurchaninov, Y. A. Kurochkin, M. G. Kurth, E. S. Kuwertz, M. Kuze, J. Kvita, T. Kwan, D. Kyriazopoulos, A. La Rosa, J. L. La Rosa Navarro, L. La Rotonda, F. La Ruffa, C. Lacasta, F. Lacava, J. Lacey, D. P. J. Lack, H. Lacker, D. Lacour, E. Ladygin, R. Lafaye, B. Laforge, T. Lagouri, S. Lai, S. Lammers, W. Lampl, E. Lançon, U. Landgraf, M. P. J. Landon, M. C. Lanfermann, V. S. Lang, J. C. Lange, R. J. Langenberg, A. J. Lankford, F. Lanni, K. Lantzsch, A. Lanza, A. Lapertosa, S. Laplace, J. F. Laporte, T. Lari, F. Lasagni Manghi, M. Lassnig, T. S. Lau, P. Laurelli, W. Lavrijsen, A. T. Law, P. Laycock, T. Lazovich, M. Lazzaroni, B. Le, O. Le Dortz, E. Le Guirriec, E. P. Le Quilleuc, M. LeBlanc, T. LeCompte, F. Ledroit-Guillon, C. A. Lee, G. R. Lee, S. C. Lee, L. Lee, B. Lefebvre, G. Lefebvre, M. Lefebvre, F. Legger, C. Leggett, G. Lehmann Miotto, X. Lei, W. A. Leight, M. A. L. Leite, R. Leitner, D. Lellouch, B. Lemmer, K. J. C. Leney, T. Lenz, B. Lenzi, R. Leone, S. Leone, C. Leonidopoulos, G. Lerner, C. Leroy, R. Les, A. A. J. Lesage, C. G. Lester, M. Levchenko, J. Levêque, D. Levin, L. J. Levinson, M. Levy, D. Lewis, B. Li, H. Li, L. Li, Q. Li, Q. Li, S. Li, X. Li, Y. Li, Z. Liang, B. Liberti, A. Liblong, K. Lie, J. Liebal, W. Liebig, A. Limosani, C. Y. Lin, K. Lin, S. C. Lin, T. H. Lin, R. A. Linck, B. E. Lindquist, A. E. Lionti, E. Lipeles, A. Lipniacka, M. Lisovyi, T. M. Liss, A. Lister, A. M. Litke, B. Liu, H. Liu, H. Liu, J. K. K. Liu, J. Liu, J. B. Liu, K. Liu, L. Liu, M. Liu, Y. L. Liu, Y. Liu, M. Livan, A. Lleres, J. Llorente Merino, S. L. Lloyd, C. Y. Lo, F. Lo Sterzo, E. M. Lobodzinska, P. Loch, F. K. Loebinger, A. Loesle, K. M. Loew, T. Lohse, K. Lohwasser, M. Lokajicek, B. A. Long, J. D. Long, R. E. Long, L. Longo, K. A. Looper, J. A. Lopez, I. Lopez Paz, A. Lopez Solis, J. Lorenz, N. Lorenzo Martinez, M. Losada, P. J. Lösel, X. Lou, A. Lounis, J. Love, P. A. Love, H. Lu, N. Lu, Y. J. Lu, H. J. Lubatti, C. Luci, A. Lucotte, C. Luedtke, F. Luehring, W. Lukas, L. Luminari, O. Lundberg, B. Lund-Jensen, M. S. Lutz, P. M. Luzi, D. Lynn, R. Lysak, E. Lytken, F. Lyu, V. Lyubushkin, H. Ma, L. L. Ma, Y. Ma, G. Maccarrone, A. Macchiolo, C. M. Macdonald, B. Maček, J. Machado Miguens, D. Madaffari, R. Madar, W. F. Mader, A. Madsen, N. Madysa, J. Maeda, S. Maeland, T. Maeno, A. S. Maevskiy, V. Magerl, C. Maiani, C. Maidantchik, T. Maier, A. Maio, O. Majersky, S. Majewski, Y. Makida, N. Makovec, B. Malaescu, Pa. Malecki, V. P. Maleev, F. Malek, U. Mallik, D. Malon, C. Malone, S. Maltezos, S. Malyukov, J. Mamuzic, G. Mancini, I. Mandić, J. Maneira, L. Manhaes de Andrade Filho, J. Manjarres Ramos, K. H. Mankinen, A. Mann, A. Manousos, B. Mansoulie, J. D. Mansour, R. Mantifel, M. Mantoani, S. Manzoni, L. Mapelli, G. Marceca, L. March, L. Marchese, G. Marchiori, M. Marcisovsky, C. A. Marin Tobon, M. Marjanovic, D. E. Marley, F. Marroquim, S. P. Marsden, Z. Marshall, M. U. F. Martensson, S. Marti-Garcia, C. B. Martin, T. A. Martin, V. J. Martin, B. Martin dit Latour, M. Martinez, V. I. Martinez Outschoorn, S. Martin-Haugh, V. S. Martoiu, A. C. Martyniuk, A. Marzin, L. Masetti, T. Mashimo, R. Mashinistov, J. Masik, A. L. Maslennikov, L. H. Mason, L. Massa, P. Mastrandrea, A. Mastroberardino, T. Masubuchi, P. Mättig, J. Maurer, S. J. Maxfield, D. A. Maximov, R. Mazini, I. Maznas, S. M. Mazza, N. C. Mc Fadden, G. Mc Goldrick, S. P. Mc Kee, A. McCarn, R. L. McCarthy, T. G. McCarthy, L. I. McClymont, E. F. McDonald, J. A. Mcfayden, G. Mchedlidze, S. J. McMahon, P. C. McNamara, C. J. McNicol, R. A. McPherson, S. Meehan, T. J. Megy, S. Mehlhase, A. Mehta, T. Meideck, K. Meier, B. Meirose, D. Melini, B. R. Mellado Garcia, J. D. Mellenthin, M. Melo, F. Meloni, A. Melzer, S. B. Menary, L. Meng, X. T. Meng, A. Mengarelli, S. Menke, E. Meoni, S. Mergelmeyer, C. Merlassino, P. Mermod, L. Merola, C. Meroni, F. S. Merritt, A. Messina, J. Metcalfe, A. S. Mete, C. Meyer, J.-P. Meyer, J. Meyer, H. Meyer Zu Theenhausen, F. Miano, R. P. Middleton, S. Miglioranzi, L. Mijović, G. Mikenberg, M. Mikestikova, M. Mikuž, M. Milesi, A. Milic, D. A. Millar, D. W. Miller, C. Mills, A. Milov, D. A. Milstead, A. A. Minaenko, Y. Minami, I. A. Minashvili, A. I. Mincer, B. Mindur, M. Mineev, Y. Minegishi, Y. Ming, L. M. Mir, A. Mirto, K. P. Mistry, T. Mitani, J. Mitrevski, V. A. Mitsou, A. Miucci, P. S. Miyagawa, A. Mizukami, J. U. Mjörnmark, T. Mkrtchyan, M. Mlynarikova, T. Moa, K. Mochizuki, P. Mogg, S. Mohapatra, S. Molander, R. Moles-Valls, M. C. Mondragon, K. Mönig, J. Monk, E. Monnier, A. Montalbano, J. Montejo Berlingen, F. Monticelli, S. Monzani, R. W. Moore, N. Morange, D. Moreno, M. Moreno Llácer, P. Morettini, S. Morgenstern, D. Mori, T. Mori, M. Morii, M. Morinaga, V. Morisbak, A. K. Morley, G. Mornacchi, J. D. Morris, L. Morvaj, P. Moschovakos, M. Mosidze, H. J. Moss, J. Moss, K. Motohashi, R. Mount, E. Mountricha, E. J. W. Moyse, S. Muanza, F. Mueller, J. Mueller, R. S. P. Mueller, D. Muenstermann, P. Mullen, G. A. Mullier, F. J. Munoz Sanchez, W. J. Murray, H. Musheghyan, M. Muškinja, A. G. Myagkov, M. Myska, B. P. Nachman, O. Nackenhorst, K. Nagai, R. Nagai, K. Nagano, Y. Nagasaka, K. Nagata, M. Nagel, E. Nagy, A. M. Nairz, Y. Nakahama, K. Nakamura, T. Nakamura, I. Nakano, R. F. Naranjo Garcia, R. Narayan, D. I. Narrias Villar, I. Naryshkin, T. Naumann, G. Navarro, R. Nayyar, H. A. Neal, P. Yu. Nechaeva, T. J. Neep, A. Negri, M. Negrini, S. Nektarijevic, C. Nellist, A. Nelson, M. E. Nelson, S. Nemecek, P. Nemethy, M. Nessi, M. S. Neubauer, M. Neumann, P. R. Newman, T. Y. Ng, Y. S. Ng, T. Nguyen Manh, R. B. Nickerson, R. Nicolaidou, J. Nielsen, N. Nikiforou, V. Nikolaenko, I. Nikolic-Audit, K. Nikolopoulos, P. Nilsson, Y. Ninomiya, A. Nisati, N. Nishu, R. Nisius, I. Nitsche, T. Nitta, T. Nobe, Y. Noguchi, M. Nomachi, I. Nomidis, M. A. Nomura, T. Nooney, M. Nordberg, N. Norjoharuddeen, O. Novgorodova, M. Nozaki, L. Nozka, K. Ntekas, E. Nurse, F. Nuti, K. O’connor, D. C. O’Neil, A. A. O’Rourke, V. O’Shea, F. G. Oakham, H. Oberlack, T. Obermann, J. Ocariz, A. Ochi, I. Ochoa, J. P. Ochoa-Ricoux, S. Oda, S. Odaka, A. Oh, S. H. Oh, C. C. Ohm, H. Ohman, H. Oide, H. Okawa, Y. Okumura, T. Okuyama, A. Olariu, L. F. Oleiro Seabra, S. A. Olivares Pino, D. Oliveira Damazio, J. Olszowska, A. Onofre, K. Onogi, P. U. E. Onyisi, H. Oppen, M. J. Oreglia, Y. Oren, D. Orestano, N. Orlando, R. S. Orr, B. Osculati, R. Ospanov, G. Otero y Garzon, H. Otono, M. Ouchrif, F. Ould-Saada, A. Ouraou, K. P. Oussoren, Q. Ouyang, M. Owen, R. E. Owen, V. E. Ozcan, N. Ozturk, K. Pachal, A. Pacheco Pages, L. Pacheco Rodriguez, C. Padilla Aranda, S. Pagan Griso, M. Paganini, F. Paige, G. Palacino, S. Palazzo, S. Palestini, M. Palka, D. Pallin, E. St. Panagiotopoulou, I. Panagoulias, C. E. Pandini, J. G. Panduro Vazquez, P. Pani, S. Panitkin, D. Pantea, L. Paolozzi, Th. D. Papadopoulou, K. Papageorgiou, A. Paramonov, D. Paredes Hernandez, A. J. Parker, M. A. Parker, K. A. Parker, F. Parodi, J. A. Parsons, U. Parzefall, V. R. Pascuzzi, J. M. Pasner, E. Pasqualucci, S. Passaggio, Fr. Pastore, S. Pataraia, J. R. Pater, T. Pauly, B. Pearson, S. Pedraza Lopez, R. Pedro, S. V. Peleganchuk, O. Penc, C. Peng, H. Peng, J. Penwell, B. S. Peralva, M. M. Perego, D. V. Perepelitsa, F. Peri, L. Perini, H. Pernegger, S. Perrella, R. Peschke, V. D. Peshekhonov, K. Peters, R. F. Y. Peters, B. A. Petersen, T. C. Petersen, E. Petit, A. Petridis, C. Petridou, P. Petroff, E. Petrolo, M. Petrov, F. Petrucci, N. E. Pettersson, A. Peyaud, R. Pezoa, F. H. Phillips, P. W. Phillips, G. Piacquadio, E. Pianori, A. Picazio, M. A. Pickering, R. Piegaia, J. E. Pilcher, A. D. Pilkington, M. Pinamonti, J. L. Pinfold, H. Pirumov, M. Pitt, L. Plazak, M.-A. Pleier, V. Pleskot, E. Plotnikova, D. Pluth, P. Podberezko, R. Poettgen, R. Poggi, L. Poggioli, I. Pogrebnyak, D. Pohl, I. Pokharel, G. Polesello, A. Poley, A. Policicchio, R. Polifka, A. Polini, C. S. Pollard, V. Polychronakos, K. Pommès, D. Ponomarenko, L. Pontecorvo, G. A. Popeneciu, D. M. Portillo Quintero, S. Pospisil, K. Potamianos, I. N. Potrap, C. J. Potter, H. Potti, T. Poulsen, J. Poveda, M. E. Pozo Astigarraga, P. Pralavorio, A. Pranko, S. Prell, D. Price, M. Primavera, S. Prince, N. Proklova, K. Prokofiev, F. Prokoshin, S. Protopopescu, J. Proudfoot, M. Przybycien, A. Puri, P. Puzo, J. Qian, G. Qin, Y. Qin, A. Quadt, M. Queitsch-Maitland, D. Quilty, S. Raddum, V. Radeka, V. Radescu, S. K. Radhakrishnan, P. Radloff, P. Rados, F. Ragusa, G. Rahal, J. A. Raine, S. Rajagopalan, C. Rangel-Smith, T. Rashid, S. Raspopov, M. G. Ratti, D. M. Rauch, F. Rauscher, S. Rave, I. Ravinovich, J. H. Rawling, M. Raymond, A. L. Read, N. P. Readioff, M. Reale, D. M. Rebuzzi, A. Redelbach, G. Redlinger, R. Reece, R. G. Reed, K. Reeves, L. Rehnisch, J. Reichert, A. Reiss, C. Rembser, H. Ren, M. Rescigno, S. Resconi, E. D. Resseguie, S. Rettie, E. Reynolds, O. L. Rezanova, P. Reznicek, R. Rezvani, R. Richter, S. Richter, E. Richter-Was, O. Ricken, M. Ridel, P. Rieck, C. J. Riegel, J. Rieger, O. Rifki, M. Rijssenbeek, A. Rimoldi, M. Rimoldi, L. Rinaldi, G. Ripellino, B. Ristić, E. Ritsch, I. Riu, F. Rizatdinova, E. Rizvi, C. Rizzi, R. T. Roberts, S. H. Robertson, A. Robichaud-Veronneau, D. Robinson, J. E. M. Robinson, A. Robson, E. Rocco, C. Roda, Y. Rodina, S. Rodriguez Bosca, A. Rodriguez Perez, D. Rodriguez Rodriguez, S. Roe, C. S. Rogan, O. Røhne, J. Roloff, A. Romaniouk, M. Romano, S. M. Romano Saez, E. Romero Adam, N. Rompotis, M. Ronzani, L. Roos, S. Rosati, K. Rosbach, P. Rose, N.-A. Rosien, E. Rossi, L. P. Rossi, J. H. N. Rosten, R. Rosten, M. Rotaru, J. Rothberg, D. Rousseau, D. Roy, A. Rozanov, Y. Rozen, X. Ruan, F. Rubbo, F. Rühr, A. Ruiz-Martinez, Z. Rurikova, N. A. Rusakovich, H. L. Russell, J. P. Rutherfoord, N. Ruthmann, E. M. Rüttinger, Y. F. Ryabov, M. Rybar, G. Rybkin, S. Ryu, A. Ryzhov, G. F. Rzehorz, A. F. Saavedra, G. Sabato, S. Sacerdoti, H. F.-W. Sadrozinski, R. Sadykov, F. Safai Tehrani, P. Saha, M. Sahinsoy, M. Saimpert, M. Saito, T. Saito, H. Sakamoto, Y. Sakurai, G. Salamanna, J. E. Salazar Loyola, D. Salek, P. H. Sales De Bruin, D. Salihagic, A. Salnikov, J. Salt, D. Salvatore, F. Salvatore, A. Salvucci, A. Salzburger, D. Sammel, D. Sampsonidis, D. Sampsonidou, J. Sánchez, V. Sanchez Martinez, A. Sanchez Pineda, H. Sandaker, R. L. Sandbach, C. O. Sander, M. Sandhoff, C. Sandoval, D. P. C. Sankey, M. Sannino, Y. Sano, A. Sansoni, C. Santoni, H. Santos, I. Santoyo Castillo, A. Sapronov, J. G. Saraiva, B. Sarrazin, O. Sasaki, K. Sato, E. Sauvan, G. Savage, P. Savard, N. Savic, C. Sawyer, L. Sawyer, J. Saxon, C. Sbarra, A. Sbrizzi, T. Scanlon, D. A. Scannicchio, J. Schaarschmidt, P. Schacht, B. M. Schachtner, D. Schaefer, L. Schaefer, R. Schaefer, J. Schaeffer, S. Schaepe, S. Schaetzel, U. Schäfer, A. C. Schaffer, D. Schaile, R. D. Schamberger, V. A. Schegelsky, D. Scheirich, F. Schenck, M. Schernau, C. Schiavi, S. Schier, L. K. Schildgen, C. Schillo, M. Schioppa, S. Schlenker, K. R. Schmidt-Sommerfeld, K. Schmieden, C. Schmitt, S. Schmitt, S. Schmitz, U. Schnoor, L. Schoeffel, A. Schoening, B. D. Schoenrock, E. Schopf, M. Schott, J. F. P. Schouwenberg, J. Schovancova, S. Schramm, N. Schuh, A. Schulte, M. J. Schultens, H.-C. Schultz-Coulon, H. Schulz, M. Schumacher, B. A. Schumm, Ph. Schune, A. Schwartzman, T. A. Schwarz, H. Schweiger, Ph. Schwemling, R. Schwienhorst, J. Schwindling, A. Sciandra, G. Sciolla, M. Scornajenghi, F. Scuri, F. Scutti, J. Searcy, P. Seema, S. C. Seidel, A. Seiden, J. M. Seixas, G. Sekhniaidze, K. Sekhon, S. J. Sekula, N. Semprini-Cesari, S. Senkin, C. Serfon, L. Serin, L. Serkin, M. Sessa, R. Seuster, H. Severini, T. Šfiligoj, F. Sforza, A. Sfyrla, E. Shabalina, N. W. Shaikh, L. Y. Shan, R. Shang, J. T. Shank, M. Shapiro, P. B. Shatalov, K. Shaw, S. M. Shaw, A. Shcherbakova, C. Y. Shehu, Y. Shen, N. Sherafati, A. D. Sherman, P. Sherwood, L. Shi, S. Shimizu, C. O. Shimmin, M. Shimojima, I. P. J. Shipsey, S. Shirabe, M. Shiyakova, J. Shlomi, A. Shmeleva, D. Shoaleh Saadi, M. J. Shochet, S. Shojaii, D. R. Shope, S. Shrestha, E. Shulga, M. A. Shupe, P. Sicho, A. M. Sickles, P. E. Sidebo, E. Sideras Haddad, O. Sidiropoulou, A. Sidoti, F. Siegert, Dj. Sijacki, J. Silva, S. B. Silverstein, V. Simak, L. Simic, S. Simion, E. Simioni, B. Simmons, M. Simon, P. Sinervo, N. B. Sinev, M. Sioli, G. Siragusa, I. Siral, S. Yu. Sivoklokov, J. Sjölin, M. B. Skinner, P. Skubic, M. Slater, T. Slavicek, M. Slawinska, K. Sliwa, R. Slovak, V. Smakhtin, B. H. Smart, J. Smiesko, N. Smirnov, S. Yu. Smirnov, Y. Smirnov, L. N. Smirnova, O. Smirnova, J. W. Smith, M. N. K. Smith, R. W. Smith, M. Smizanska, K. Smolek, A. A. Snesarev, I. M. Snyder, S. Snyder, R. Sobie, F. Socher, A. Soffer, A. Søgaard, D. A. Soh, G. Sokhrannyi, C. A. Solans Sanchez, M. Solar, E. Yu. Soldatov, U. Soldevila, A. A. Solodkov, A. Soloshenko, O. V. Solovyanov, V. Solovyev, P. Sommer, H. Son, A. Sopczak, D. Sosa, C. L. Sotiropoulou, S. Sottocornola, R. Soualah, A. M. Soukharev, D. South, B. C. Sowden, S. Spagnolo, M. Spalla, M. Spangenberg, F. Spanò, D. Sperlich, F. Spettel, T. M. Spieker, R. Spighi, G. Spigo, L. A. Spiller, M. Spousta, R. D. St. Denis, A. Stabile, R. Stamen, S. Stamm, E. Stanecka, R. W. Stanek, C. Stanescu, M. M. Stanitzki, B. S. Stapf, S. Stapnes, E. A. Starchenko, G. H. Stark, J. Stark, S. H Stark, P. Staroba, P. Starovoitov, S. Stärz, R. Staszewski, M. Stegler, P. Steinberg, B. Stelzer, H. J. Stelzer, O. Stelzer-Chilton, H. Stenzel, T. J. Stevenson, G. A. Stewart, M. C. Stockton, M. Stoebe, G. Stoicea, P. Stolte, S. Stonjek, A. R. Stradling, A. Straessner, M. E. Stramaglia, J. Strandberg, S. Strandberg, M. Strauss, P. Strizenec, R. Ströhmer, D. M. Strom, R. Stroynowski, A. Strubig, S. A. Stucci, B. Stugu, N. A. Styles, D. Su, J. Su, S. Suchek, Y. Sugaya, M. Suk, V. V. Sulin, D. M. S. Sultan, S. Sultansoy, T. Sumida, S. Sun, X. Sun, K. Suruliz, C. J. E. Suster, M. R. Sutton, S. Suzuki, M. Svatos, M. Swiatlowski, S. P. Swift, I. Sykora, T. Sykora, D. Ta, K. Tackmann, J. Taenzer, A. Taffard, R. Tafirout, E. Tahirovic, N. Taiblum, H. Takai, R. Takashima, E. H. Takasugi, K. Takeda, T. Takeshita, Y. Takubo, M. Talby, A. A. Talyshev, J. Tanaka, M. Tanaka, R. Tanaka, S. Tanaka, R. Tanioka, B. B. Tannenwald, S. Tapia Araya, S. Tapprogge, S. Tarem, G. F. Tartarelli, P. Tas, M. Tasevsky, T. Tashiro, E. Tassi, A. Tavares Delgado, Y. Tayalati, A. C. Taylor, A. J. Taylor, G. N. Taylor, P. T. E. Taylor, W. Taylor, P. Teixeira-Dias, D. Temple, H. Ten Kate, P. K. Teng, J. J. Teoh, F. Tepel, S. Terada, K. Terashi, J. Terron, S. Terzo, M. Testa, R. J. Teuscher, S. J. Thais, T. Theveneaux-Pelzer, F. Thiele, J. P. Thomas, J. Thomas-Wilsker, P. D. Thompson, A. S. Thompson, L. A. Thomsen, E. Thomson, Y. Tian, M. J. Tibbetts, R. E. Ticse Torres, V. O. Tikhomirov, Yu. A. Tikhonov, S. Timoshenko, P. Tipton, S. Tisserant, K. Todome, S. Todorova-Nova, S. Todt, J. Tojo, S. Tokár, K. Tokushuku, E. Tolley, L. Tomlinson, M. Tomoto, L. Tompkins, K. Toms, B. Tong, P. Tornambe, E. Torrence, H. Torres, E. Torró Pastor, J. Toth, F. Touchard, D. R. Tovey, C. J. Treado, T. Trefzger, F. Tresoldi, A. Tricoli, I. M. Trigger, S. Trincaz-Duvoid, M. F. Tripiana, W. Trischuk, B. Trocmé, A. Trofymov, C. Troncon, M. Trottier-McDonald, M. Trovatelli, L. Truong, M. Trzebinski, A. Trzupek, K. W. Tsang, J. C.-L. Tseng, P. V. Tsiareshka, N. Tsirintanis, S. Tsiskaridze, V. Tsiskaridze, E. G. Tskhadadze, I. I. Tsukerman, V. Tsulaia, S. Tsuno, D. Tsybychev, Y. Tu, A. Tudorache, V. Tudorache, T. T. Tulbure, A. N. Tuna, S. Turchikhin, D. Turgeman, I. Turk Cakir, R. Turra, P. M. Tuts, G. Ucchielli, I. Ueda, M. Ughetto, F. Ukegawa, G. Unal, A. Undrus, G. Unel, F. C. Ungaro, Y. Unno, K. Uno, C. Unverdorben, J. Urban, P. Urquijo, P. Urrejola, G. Usai, J. Usui, L. Vacavant, V. Vacek, B. Vachon, K. O. H. Vadla, A. Vaidya, C. Valderanis, E. Valdes Santurio, M. Valente, S. Valentinetti, A. Valero, L. Valéry, S. Valkar, A. Vallier, J. A. Valls Ferrer, W. Van Den Wollenberg, H. van der Graaf, P. van Gemmeren, J. Van Nieuwkoop, I. van Vulpen, M. C. van Woerden, M. Vanadia, W. Vandelli, A. Vaniachine, P. Vankov, G. Vardanyan, R. Vari, E. W. Varnes, C. Varni, T. Varol, D. Varouchas, A. Vartapetian, K. E. Varvell, J. G. Vasquez, G. A. Vasquez, F. Vazeille, D. Vazquez Furelos, T. Vazquez Schroeder, J. Veatch, V. Veeraraghavan, L. M. Veloce, F. Veloso, S. Veneziano, A. Ventura, M. Venturi, N. Venturi, A. Venturini, V. Vercesi, M. Verducci, W. Verkerke, A. T. Vermeulen, J. C. Vermeulen, M. C. Vetterli, N. Viaux Maira, O. Viazlo, I. Vichou, T. Vickey, O. E. Vickey Boeriu, G. H. A. Viehhauser, S. Viel, L. Vigani, M. Villa, M. Villaplana Perez, E. Vilucchi, M. G. Vincter, V. B. Vinogradov, A. Vishwakarma, C. Vittori, I. Vivarelli, S. Vlachos, M. Vogel, P. Vokac, G. Volpi, H. von der Schmitt, E. von Toerne, V. Vorobel, K. Vorobev, M. Vos, R. Voss, J. H. Vossebeld, N. Vranjes, M. Vranjes Milosavljevic, V. Vrba, M. Vreeswijk, R. Vuillermet, I. Vukotic, P. Wagner, W. Wagner, J. Wagner-Kuhr, H. Wahlberg, S. Wahrmund, K. Wakamiya, J. Walder, R. Walker, W. Walkowiak, V. Wallangen, C. Wang, C. Wang, F. Wang, H. Wang, H. Wang, J. Wang, J. Wang, Q. Wang, R.-J. Wang, R. Wang, S. M. Wang, T. Wang, W. Wang, W. Wang, Z. Wang, C. Wanotayaroj, A. Warburton, C. P. Ward, D. R. Wardrope, A. Washbrook, P. M. Watkins, A. T. Watson, M. F. Watson, G. Watts, S. Watts, B. M. Waugh, A. F. Webb, S. Webb, M. S. Weber, S. M. Weber, S. W. Weber, S. A. Weber, J. S. Webster, A. R. Weidberg, B. Weinert, J. Weingarten, M. Weirich, C. Weiser, H. Weits, P. S. Wells, T. Wenaus, T. Wengler, S. Wenig, N. Wermes, M. D. Werner, P. Werner, M. Wessels, T. D. Weston, K. Whalen, N. L. Whallon, A. M. Wharton, A. S. White, A. White, M. J. White, R. White, D. Whiteson, B. W. Whitmore, F. J. Wickens, W. Wiedenmann, M. Wielers, C. Wiglesworth, L. A. M. Wiik-Fuchs, A. Wildauer, F. Wilk, H. G. Wilkens, H. H. Williams, S. Williams, C. Willis, S. Willocq, J. A. Wilson, I. Wingerter-Seez, E. Winkels, F. Winklmeier, O. J. Winston, B. T. Winter, M. Wittgen, M. Wobisch, A. Wolf, T. M. H. Wolf, R. Wolff, M. W. Wolter, H. Wolters, V. W. S. Wong, N. L. Woods, S. D. Worm, B. K. Wosiek, J. Wotschack, K. W. Wozniak, M. Wu, S. L. Wu, X. Wu, Y. Wu, T. R. Wyatt, B. M. Wynne, S. Xella, Z. Xi, L. Xia, D. Xu, L. Xu, T. Xu, W. Xu, B. Yabsley, S. Yacoob, D. Yamaguchi, Y. Yamaguchi, A. Yamamoto, S. Yamamoto, T. Yamanaka, F. Yamane, M. Yamatani, T. Yamazaki, Y. Yamazaki, Z. Yan, H. Yang, H. Yang, Y. Yang, Z. Yang, W.-M. Yao, Y. C. Yap, Y. Yasu, E. Yatsenko, K. H. Yau Wong, J. Ye, S. Ye, I. Yeletskikh, E. Yigitbasi, E. Yildirim, K. Yorita, K. Yoshihara, C. Young, C. J. S. Young, J. Yu, J. Yu, S. P. Y. Yuen, I. Yusuff, B. Zabinski, G. Zacharis, R. Zaidan, A. M. Zaitsev, N. Zakharchuk, J. Zalieckas, A. Zaman, S. Zambito, D. Zanzi, C. Zeitnitz, G. Zemaityte, A. Zemla, J. C. Zeng, Q. Zeng, O. Zenin, T. Ženiš, D. Zerwas, D. Zhang, D. Zhang, F. Zhang, G. Zhang, H. Zhang, J. Zhang, L. Zhang, L. Zhang, M. Zhang, P. Zhang, R. Zhang, R. Zhang, X. Zhang, Y. Zhang, Z. Zhang, X. Zhao, Y. Zhao, Z. Zhao, A. Zhemchugov, B. Zhou, C. Zhou, L. Zhou, M. Zhou, M. Zhou, N. Zhou, Y. Zhou, C. G. Zhu, H. Zhu, J. Zhu, Y. Zhu, X. Zhuang, K. Zhukov, A. Zibell, D. Zieminska, N. I. Zimine, C. Zimmermann, S. Zimmermann, Z. Zinonos, M. Zinser, M. Ziolkowski, L. Živković, G. Zobernig, A. Zoccoli, R. Zou, M. zur Nedden, L. Zwalinski, ATLAS Collaboration

**Affiliations:** 1 Department of Physics, University of Adelaide, Adelaide, Australia; 2 Physics Department, SUNY Albany, Albany, NY USA; 3 Department of Physics, University of Alberta, Edmonton, AB Canada; 4 Department of Physics, Ankara University, Ankara, Turkey; 5 Istanbul Aydin University, Istanbul, Turkey; 6 Division of Physics, TOBB University of Economics and Technology, Ankara, Turkey; 7 LAPP, CNRS/IN2P3 and Université Savoie Mont Blanc, Annecy-le-Vieux, France; 8 High Energy Physics Division, Argonne National Laboratory, Argonne, IL USA; 9 Department of Physics, University of Arizona, Tucson, AZ USA; 10 Department of Physics, The University of Texas at Arlington, Arlington, TX USA; 11 Physics Department, National and Kapodistrian University of Athens, Athens, Greece; 12 Physics Department, National Technical University of Athens, Zografou, Greece; 13 Department of Physics, The University of Texas at Austin, Austin, TX USA; 14 Institute of Physics, Azerbaijan Academy of Sciences, Baku, Azerbaijan; 15 Institut de Física d’Altes Energies (IFAE), The Barcelona Institute of Science and Technology, Barcelona, Spain; 16 Institute of Physics, University of Belgrade, Belgrade, Serbia; 17 Department for Physics and Technology, University of Bergen, Bergen, Norway; 18 Physics Division, Lawrence Berkeley National Laboratory, University of California, Berkeley, CA USA; 19 Department of Physics, Humboldt University, Berlin, Germany; 20 Albert Einstein Center for Fundamental Physics, Laboratory for High Energy Physics, University of Bern, Bern, Switzerland; 21 School of Physics and Astronomy, University of Birmingham, Birmingham, UK; 22 Department of Physics, Bogazici University, Istanbul, Turkey; 23 Department of Physics Engineering, Gaziantep University, Gaziantep, Turkey; 24 Faculty of Engineering and Natural Sciences, Istanbul Bilgi University, Istanbul, Turkey; 25 Faculty of Engineering and Natural Sciences, Bahcesehir University, Istanbul, Turkey; 26 Centro de Investigaciones, Universidad Antonio Narino, Bogota, Colombia; 27 INFN Sezione di Bologna, Bologna, Italy; 28 Dipartimento di Fisica e Astronomia, Università di Bologna, Bologna, Italy; 29 Physikalisches Institut, University of Bonn, Bonn, Germany; 30 Department of Physics, Boston University, Boston, MA USA; 31 Department of Physics, Brandeis University, Waltham, MA USA; 32 Universidade Federal do Rio De Janeiro COPPE/EE/IF, Rio de Janeiro, Brazil; 33 Electrical Circuits Department, Federal University of Juiz de Fora (UFJF), Juiz de Fora, Brazil; 34 Federal University of Sao Joao del Rei (UFSJ), Sao Joao del Rei, Brazil; 35 Instituto de Fisica, Universidade de Sao Paulo, Sao Paulo, Brazil; 36 Physics Department, Brookhaven National Laboratory, Upton, NY USA; 37 Transilvania University of Brasov, Brasov, Romania; 38 Horia Hulubei National Institute of Physics and Nuclear Engineering, Bucharest, Romania; 39 Department of Physics, Alexandru Ioan Cuza University of Iasi, Iasi, Romania; 40 Physics Department, National Institute for Research and Development of Isotopic and Molecular Technologies, Cluj-Napoca, Romania; 41 University Politehnica Bucharest, Bucharest, Romania; 42 West University in Timisoara, Timisoara, Romania; 43 Departamento de Física, Universidad de Buenos Aires, Buenos Aires, Argentina; 44 Cavendish Laboratory, University of Cambridge, Cambridge, UK; 45 Department of Physics, Carleton University, Ottawa, ON Canada; 46 CERN, Geneva, Switzerland; 47 Enrico Fermi Institute, University of Chicago, Chicago, IL USA; 48 Departamento de Física, Pontificia Universidad Católica de Chile, Santiago, Chile; 49 Departamento de Física, Universidad Técnica Federico Santa María, Valparaíso, Chile; 50 Institute of High Energy Physics, Chinese Academy of Sciences, Beijing, China; 51 Department of Physics, Nanjing University, Nanjing, Jiangsu China; 52 Physics Department, Tsinghua University, Beijing, 100084 China; 53 University of Chinese Academy of Science (UCAS), Beijing, China; 54 Department of Modern Physics and State Key Laboratory of Particle Detection and Electronics, University of Science and Technology of China, Hefei, Anhui China; 55 School of Physics, Shandong University, Jinan, Shandong China; 56 Department of Physics and Astronomy, Key Laboratory for Particle Physics, Astrophysics and Cosmology, Ministry of Education, Shanghai Key Laboratory for Particle Physics and Cosmology, Shanghai Jiao Tong University, Tsung-Dao Lee Institute, Shanghai, China; 57 Université Clermont Auvergne, CNRS/IN2P3, LPC, Clermont-Ferrand, France; 58 Nevis Laboratory, Columbia University, Irvington, NY USA; 59 Niels Bohr Institute, University of Copenhagen, Copenhagen, Denmark; 60 INFN Gruppo Collegato di Cosenza, Laboratori Nazionali di Frascati, Frascati, Italy; 61 Dipartimento di Fisica, Università della Calabria, Rende, Italy; 62 Faculty of Physics and Applied Computer Science, AGH University of Science and Technology, Kraków, Poland; 63 Marian Smoluchowski Institute of Physics, Jagiellonian University, Kraków, Poland; 64 Institute of Nuclear Physics, Polish Academy of Sciences, Kraków, Poland; 65 Physics Department, Southern Methodist University, Dallas, TX USA; 66 Physics Department, University of Texas at Dallas, Richardson, TX USA; 67 DESY, Hamburg and Zeuthen, Germany; 68 Lehrstuhl für Experimentelle Physik IV, Technische Universität Dortmund, Dortmund, Germany; 69 Institut für Kern- und Teilchenphysik, Technische Universität Dresden, Dresden, Germany; 70 Department of Physics, Duke University, Durham, NC USA; 71 SUPA-School of Physics and Astronomy, University of Edinburgh, Edinburgh, UK; 72 INFN e Laboratori Nazionali di Frascati, Frascati, Italy; 73 Fakultät für Mathematik und Physik, Albert-Ludwigs-Universität, Freiburg, Germany; 74 Departement de Physique Nucleaire et Corpusculaire, Université de Genève, Geneva, Switzerland; 75 INFN Sezione di Genova, Genoa, Italy; 76 Dipartimento di Fisica, Università di Genova, Genoa, Italy; 77 E. Andronikashvili Institute of Physics, Iv. Javakhishvili Tbilisi State University, Tbilisi, Georgia; 78 High Energy Physics Institute, Tbilisi State University, Tbilisi, Georgia; 79 II Physikalisches Institut, Justus-Liebig-Universität Giessen, Giessen, Germany; 80 SUPA-School of Physics and Astronomy, University of Glasgow, Glasgow, UK; 81 II Physikalisches Institut, Georg-August-Universität, Göttingen, Germany; 82 Laboratoire de Physique Subatomique et de Cosmologie, Université Grenoble-Alpes, CNRS/IN2P3, Grenoble, France; 83 Laboratory for Particle Physics and Cosmology, Harvard University, Cambridge, MA USA; 84 Kirchhoff-Institut für Physik, Ruprecht-Karls-Universität Heidelberg, Heidelberg, Germany; 85 Physikalisches Institut, Ruprecht-Karls-Universität Heidelberg, Heidelberg, Germany; 86 Faculty of Applied Information Science, Hiroshima Institute of Technology, Hiroshima, Japan; 87 Department of Physics, The Chinese University of Hong Kong, Shatin, NT Hong Kong; 88 Department of Physics, The University of Hong Kong, Hong Kong, China; 89 Department of Physics, Institute for Advanced Study, The Hong Kong University of Science and Technology, Clear Water Bay, Kowloon, Hong Kong, China; 90 Department of Physics, National Tsing Hua University, Hsinchu, Taiwan; 91 Department of Physics, Indiana University, Bloomington, IN USA; 92 Institut für Astro- und Teilchenphysik, Leopold-Franzens-Universität, Innsbruck, Austria; 93 University of Iowa, Iowa City, IA USA; 94 Department of Physics and Astronomy, Iowa State University, Ames, IA USA; 95 Joint Institute for Nuclear Research, JINR Dubna, Dubna, Russia; 96 KEK, High Energy Accelerator Research Organization, Tsukuba, Japan; 97 Graduate School of Science, Kobe University, Kobe, Japan; 98 Faculty of Science, Kyoto University, Kyoto, Japan; 99 Kyoto University of Education, Kyoto, Japan; 100 Research Center for Advanced Particle Physics and Department of Physics, Kyushu University, Fukuoka, Japan; 101 Instituto de Física La Plata, Universidad Nacional de La Plata and CONICET, La Plata, Argentina; 102 Physics Department, Lancaster University, Lancaster, UK; 103 INFN Sezione di Lecce, Lecce, Italy; 104 Dipartimento di Matematica e Fisica, Università del Salento, Lecce, Italy; 105 Oliver Lodge Laboratory, University of Liverpool, Liverpool, UK; 106 Department of Experimental Particle Physics, Jožef Stefan Institute and Department of Physics, University of Ljubljana, Ljubljana, Slovenia; 107 School of Physics and Astronomy, Queen Mary University of London, London, UK; 108 Department of Physics, Royal Holloway University of London, Surrey, UK; 109 Department of Physics and Astronomy, University College London, London, UK; 110 Louisiana Tech University, Ruston, LA USA; 111 Laboratoire de Physique Nucléaire et de Hautes Energies, UPMC and Université Paris-Diderot and CNRS/IN2P3, Paris, France; 112 Fysiska institutionen, Lunds universitet, Lund, Sweden; 113 Departamento de Fisica Teorica C-15, Universidad Autonoma de Madrid, Madrid, Spain; 114 Institut für Physik, Universität Mainz, Mainz, Germany; 115 School of Physics and Astronomy, University of Manchester, Manchester, UK; 116 CPPM, Aix-Marseille Université and CNRS/IN2P3, Marseille, France; 117 Department of Physics, University of Massachusetts, Amherst, MA USA; 118 Department of Physics, McGill University, Montreal, QC Canada; 119 School of Physics, University of Melbourne, Victoria, Australia; 120 Department of Physics, The University of Michigan, Ann Arbor, MI USA; 121 Department of Physics and Astronomy, Michigan State University, East Lansing, MI USA; 122 INFN Sezione di Milano, Milan, Italy; 123 Dipartimento di Fisica, Università di Milano, Milan, Italy; 124 B.I. Stepanov Institute of Physics, National Academy of Sciences of Belarus, Minsk, Republic of Belarus; 125 Research Institute for Nuclear Problems of Byelorussian State University, Minsk, Republic of Belarus; 126 Group of Particle Physics, University of Montreal, Montreal, QC Canada; 127 P.N. Lebedev Physical Institute of the Russian Academy of Sciences, Moscow, Russia; 128 Institute for Theoretical and Experimental Physics (ITEP), Moscow, Russia; 129 National Research Nuclear University MEPhI, Moscow, Russia; 130 D.V. Skobeltsyn Institute of Nuclear Physics, M.V. Lomonosov Moscow State University, Moscow, Russia; 131 Fakultät für Physik, Ludwig-Maximilians-Universität München, Munich, Germany; 132 Max-Planck-Institut für Physik (Werner-Heisenberg-Institut), Munich, Germany; 133 Nagasaki Institute of Applied Science, Nagasaki, Japan; 134 Graduate School of Science and Kobayashi-Maskawa Institute, Nagoya University, Nagoya, Japan; 135 INFN Sezione di Napoli, Naples, Italy; 136 Dipartimento di Fisica, Università di Napoli, Naples, Italy; 137 Department of Physics and Astronomy, University of New Mexico, Albuquerque, NM USA; 138 Institute for Mathematics, Astrophysics and Particle Physics, Radboud University Nijmegen/Nikhef, Nijmegen, The Netherlands; 139 Nikhef National Institute for Subatomic Physics, University of Amsterdam, Amsterdam, The Netherlands; 140 Department of Physics, Northern Illinois University, DeKalb, IL USA; 141 Budker Institute of Nuclear Physics, SB RAS, Novosibirsk, Russia; 142 Department of Physics, New York University, New York, NY USA; 143 Ohio State University, Columbus, OH USA; 144 Faculty of Science, Okayama University, Okayama, Japan; 145 Homer L. Dodge Department of Physics and Astronomy, University of Oklahoma, Norman, OK USA; 146 Department of Physics, Oklahoma State University, Stillwater, OK USA; 147 Palacký University, RCPTM, Olomouc, Czech Republic; 148 Center for High Energy Physics, University of Oregon, Eugene, OR USA; 149 LAL, Univ. Paris-Sud, CNRS/IN2P3, Université Paris-Saclay, Orsay, France; 150 Graduate School of Science, Osaka University, Osaka, Japan; 151 Department of Physics, University of Oslo, Oslo, Norway; 152 Department of Physics, Oxford University, Oxford, UK; 153 INFN Sezione di Pavia, Pavia, Italy; 154 Dipartimento di Fisica, Università di Pavia, Pavia, Italy; 155 Department of Physics, University of Pennsylvania, Philadelphia, PA USA; 156 National Research Centre “Kurchatov Institute” B.P. Konstantinov Petersburg Nuclear Physics Institute, St. Petersburg, Russia; 157 INFN Sezione di Pisa, Pisa, Italy; 158 Dipartimento di Fisica E. Fermi, Università di Pisa, Pisa, Italy; 159 Department of Physics and Astronomy, University of Pittsburgh, Pittsburgh, PA USA; 160 Laboratório de Instrumentação e Física Experimental de Partículas-LIP, Lisbon, Portugal; 161 Faculdade de Ciências, Universidade de Lisboa, Lisbon, Portugal; 162 Department of Physics, University of Coimbra, Coimbra, Portugal; 163 Centro de Física Nuclear da Universidade de Lisboa, Lisbon, Portugal; 164 Departamento de Fisica, Universidade do Minho, Braga, Portugal; 165 Departamento de Fisica Teorica y del Cosmos, Universidad de Granada, Granada, Spain; 166 Dep Fisica and CEFITEC of Faculdade de Ciencias e Tecnologia, Universidade Nova de Lisboa, Caparica, Portugal; 167 Institute of Physics, Academy of Sciences of the Czech Republic, Prague, Czech Republic; 168 Czech Technical University in Prague, Prague, Czech Republic; 169 Faculty of Mathematics and Physics, Charles University, Prague, Czech Republic; 170 State Research Center Institute for High Energy Physics (Protvino), NRC KI, Protvino, Russia; 171 Particle Physics Department, Rutherford Appleton Laboratory, Didcot, UK; 172 INFN Sezione di Roma, Rome, Italy; 173 Dipartimento di Fisica, Sapienza Università di Roma, Rome, Italy; 174 INFN Sezione di Roma Tor Vergata, Rome, Italy; 175 Dipartimento di Fisica, Università di Roma Tor Vergata, Rome, Italy; 176 INFN Sezione di Roma Tre, Rome, Italy; 177 Dipartimento di Matematica e Fisica, Università Roma Tre, Rome, Italy; 178 Faculté des Sciences Ain Chock, Réseau Universitaire de Physique des Hautes Energies-Université Hassan II, Casablanca, Morocco; 179 Centre National de l’Energie des Sciences Techniques Nucleaires, Rabat, Morocco; 180 Faculté des Sciences Semlalia, Université Cadi Ayyad, LPHEA-Marrakech, Marrakech, Morocco; 181 Faculté des Sciences, Université Mohamed Premier and LPTPM, Oujda, Morocco; 182 Faculté des Sciences, Université Mohammed V, Rabat, Morocco; 183 DSM/IRFU (Institut de Recherches sur les Lois Fondamentales de l’Univers), CEA Saclay (Commissariat à l’Energie Atomique et aux Energies Alternatives), Gif-sur-Yvette, France; 184 Santa Cruz Institute for Particle Physics, University of California Santa Cruz, Santa Cruz, CA USA; 185 Department of Physics, University of Washington, Seattle, WA USA; 186 Department of Physics and Astronomy, University of Sheffield, Sheffield, UK; 187 Department of Physics, Shinshu University, Nagano, Japan; 188 Department Physik, Universität Siegen, Siegen, Germany; 189 Department of Physics, Simon Fraser University, Burnaby, BC Canada; 190 SLAC National Accelerator Laboratory, Stanford, CA USA; 191 Faculty of Mathematics, Physics and Informatics, Comenius University, Bratislava, Slovak Republic; 192 Department of Subnuclear Physics, Institute of Experimental Physics of the Slovak Academy of Sciences, Kosice, Slovak Republic; 193 Department of Physics, University of Cape Town, Cape Town, South Africa; 194 Department of Physics, University of Johannesburg, Johannesburg, South Africa; 195 School of Physics, University of the Witwatersrand, Johannesburg, South Africa; 196 Department of Physics, Stockholm University, Stockholm, Sweden; 197 The Oskar Klein Centre, Stockholm, Sweden; 198 Physics Department, Royal Institute of Technology, Stockholm, Sweden; 199 Departments of Physics and Astronomy and Chemistry, Stony Brook University, Stony Brook, NY USA; 200 Department of Physics and Astronomy, University of Sussex, Brighton, UK; 201 School of Physics, University of Sydney, Sydney, Australia; 202 Institute of Physics, Academia Sinica, Taipei, Taiwan; 203 Department of Physics, Technion: Israel Institute of Technology, Haifa, Israel; 204 Raymond and Beverly Sackler School of Physics and Astronomy, Tel Aviv University, Tel Aviv, Israel; 205 Department of Physics, Aristotle University of Thessaloniki, Thessaloniki, Greece; 206 International Center for Elementary Particle Physics and Department of Physics, The University of Tokyo, Tokyo, Japan; 207 Graduate School of Science and Technology, Tokyo Metropolitan University, Tokyo, Japan; 208 Department of Physics, Tokyo Institute of Technology, Tokyo, Japan; 209 Tomsk State University, Tomsk, Russia; 210 Department of Physics, University of Toronto, Toronto, ON Canada; 211 INFN-TIFPA, Trento, Italy; 212 University of Trento, Trento, Italy; 213 TRIUMF, Vancouver, BC Canada; 214 Department of Physics and Astronomy, York University, Toronto, ON Canada; 215 Faculty of Pure and Applied Sciences, and Center for Integrated Research in Fundamental Science and Engineering, University of Tsukuba, Tsukuba, Japan; 216 Department of Physics and Astronomy, Tufts University, Medford, MA USA; 217 Department of Physics and Astronomy, University of California Irvine, Irvine, CA USA; 218 INFN Gruppo Collegato di Udine, Sezione di Trieste, Udine, Italy; 219 ICTP, Trieste, Italy; 220 Dipartimento di Chimica, Fisica e Ambiente, Università di Udine, Udine, Italy; 221 Department of Physics and Astronomy, University of Uppsala, Uppsala, Sweden; 222 Department of Physics, University of Illinois, Urbana, IL USA; 223 Instituto de Fisica Corpuscular (IFIC), Centro Mixto Universidad de Valencia-CSIC, Valencia, Spain; 224 Department of Physics, University of British Columbia, Vancouver, BC Canada; 225 Department of Physics and Astronomy, University of Victoria, Victoria, BC Canada; 226 Department of Physics, University of Warwick, Coventry, UK; 227 Waseda University, Tokyo, Japan; 228 Department of Particle Physics, The Weizmann Institute of Science, Rehovot, Israel; 229 Department of Physics, University of Wisconsin, Madison, WI USA; 230 Fakultät für Physik und Astronomie, Julius-Maximilians-Universität, Würzburg, Germany; 231 Fakultät für Mathematik und Naturwissenschaften, Fachgruppe Physik, Bergische Universität Wuppertal, Wuppertal, Germany; 232 Department of Physics, Yale University, New Haven, CT USA; 233 Yerevan Physics Institute, Yerevan, Armenia; 234 Centre de Calcul de l’Institut National de Physique Nucléaire et de Physique des Particules (IN2P3), Villeurbanne, France; 235 Academia Sinica Grid Computing, Institute of Physics, Academia Sinica, Taipei, Taiwan; 236 CERN, 1211 Geneva 23, Switzerland

## Abstract

A search for weakly interacting massive dark-matter particles produced in association with bottom or top quarks is presented. Final states containing third-generation quarks and missing transverse momentum are considered. The analysis uses $36.1 \; \mathrm {fb}^{-1}$ of proton–proton collision data recorded by the ATLAS experiment at $\sqrt{s}=13$ TeV in 2015 and 2016. No significant excess of events above the estimated backgrounds is observed. The results are interpreted in the framework of simplified models of spin-0 dark-matter mediators. For colour-neutral spin-0 mediators produced in association with top quarks and decaying into a pair of dark-matter particles, mediator masses below 50 GeV are excluded assuming a dark-matter candidate mass of 1 GeV and unitary couplings. For scalar and pseudoscalar mediators produced in association with bottom quarks, the search sets limits on the production cross-section of 300 times the predicted rate for mediators with masses between 10 and $50\; \hbox {GeV}$ and assuming a dark-matter mass of $1 \;\hbox {GeV}$ and unitary coupling. Constraints on colour-charged scalar simplified models are also presented. Assuming a dark-matter particle mass of $35\; \hbox {GeV}$, mediator particles with mass below $1.1\; \hbox {TeV}$ are excluded for couplings yielding a dark-matter relic density consistent with measurements.

## Introduction

Astrophysical observations have provided compelling evidence for the existence of a non-baryonic dark component of the universe: dark matter (DM) [1, 2]. The currently most accurate, although somewhat indirect, determination of DM abundance comes from global fits of cosmological parameters to a variety of observations [3, 4], while the nature of DM remains largely unknown. One of the candidates for a DM particle is a weakly interacting massive particle (WIMP) [5]. At the large hadron collider (LHC), one can search for WIMP DM ($\chi$) pair production in *pp* collisions. WIMP DM would not be detected and its production leads to signatures with missing transverse momentum. Searches for the production of DM in association with Standard Model (SM) particles have been performed at the LHC [6–12].

Recently proposed simplified benchmark models for DM production assume the existence of a mediator particle which couples both to the SM and to the dark sector [13–15]. The searches presented in this paper focus on the case of a fermionic DM particle produced through the exchange of a spin-0 mediator, which can be either a colour-neutral scalar or pseudoscalar particle (denoted by $\phi$ or *a*, respectively) or a colour-charged scalar mediator ($\phi _b$). The couplings of the mediator to the SM fermions are severely restricted by precision flavour measurements. An ansatz that automatically relaxes these constraints is Minimal Flavour Violation [16]. This assumption implies that the interaction between any new neutral spin-0 state and SM matter is proportional to the fermion masses via Yukawa-type couplings.^1^ It follows that colour-neutral mediators would be sizeably produced through loop-induced gluon fusion or in association with heavy-flavour quarks. The characteristic signature used to search for the former process is a high transverse momentum jet recoiling against missing transverse momentum [7, 11].

**Fig. 1:**
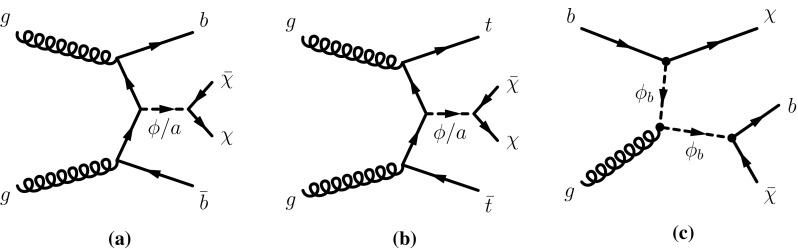
Representative diagrams at the lowest order for spin-0 mediator associated production with top and bottom quarks: **a** colour-neutral spin-0 mediator associated production with bottom quarks $b\bar{b}$ +$\phi /a$; **b** colour-neutral spin-0 mediator associated production with top quarks $t\bar{t}$ +$\phi /a$; **c** colour-charged scalar mediator model decaying into a bottom quark and a DM particle $b$-FDM

This paper focuses on dark matter produced in association with heavy flavour (top and bottom) quarks. These final states were addressed by the CMS Collaboration in Ref. [17]. For signatures with two top quarks ($t\bar{t}$ +$\phi /a$), final states where both *W* bosons decay into hadrons or both *W* bosons decay into leptons are considered in this paper. They are referred to as fully hadronic and dileptonic $t\bar{t}$ decays, respectively. Searches in final-state events characterised by fully hadronic or dileptonic top-quark pairs have been carried out targeting supersymmetric partners of the top quarks [18, 19]. Due to the different kinematics of the events under study, those searches are not optimal for the DM models considered in this paper. The search in the channel where one *W* boson decays into hadrons and one *W* boson decays into leptons (semileptonic $t\bar{t}$ decays) is presented together with the searches for top squarks in the same channel [20]. Signatures with bottom quarks in the final state are denoted $b\bar{b}$ +$\phi /a$ in the following. Representative diagrams for tree-level production of these models are shown in Fig. 1a, b. Processes with similar kinematic properties might also occur in two-Higgs-doublet models [21]. Following the notation of Ref. [14], the model has four parameters: the mass of the mediator $m_\phi$ or $m_a$, the DM mass $m_\chi$, the DM–mediator coupling $g_\chi$, and the flavour-universal SM–mediator coupling $g_q$. The mediator width is assumed to be the minimal width, which is the one calculated from the masses and couplings assumed by the model [13]. The mediator can decay into SM particles or into DM particles. This search is sensitive to decays of the mediator into a pair of DM particles. Off-shell DM production is also taken into account. The effective production cross-section of DM particles at *pp* colliders is a function of the production cross-section of the mediator, depending on $g_q$, and on the branching ratio for the mediator to decay into a pair of DM particles, which is a function of $g_q$ and $g_\chi$ [13]. The cross-section for DM production is therefore proportional to the squared product of the couplings ($g_q\;\cdot g_\chi$)$^2$, and an additional assumption of $g_q = g_\chi = g$ is made to reduce the number of parameters. Since the cross-section of annihilation and scattering from nucleons has the same functional dependence on the couplings, the same assumption is made when the results are compared to non-collider experiments.

The second category of models considered in this search is the case of colour-charged scalar mediators [22]. The model assumes bottom-flavoured dark matter ($b$-FDM) and was proposed to explain the excess of gamma rays from the galactic centre observed by the Fermi Gamma-ray Space Telescope, if this excess is to be interpreted as a signal for DM annihilation [23], while alternative conjectures without DM are also discussed [24]. A representative diagram for the production of this signal is shown in Fig. 1c. In this model, a new scalar field, $\phi _b$, mediates the interaction between DM and quarks. Dark matter is assumed to be the lightest Dirac fermion that belongs to a flavour-triplet coupling to right-handed, down-type quarks. The cosmological DM is the third component of the triplet and couples preferentially to bottom quarks. It explains the galactic-centre excess if a DM mass around $35\;\hbox {GeV}$ is assumed. The other Dirac fermions in the flavour-triplet are heavy and couple weakly, and are therefore neglected. The $b$-FDM model has three parameters: the mediator and the DM masses ($m(\phi _b)$ and $m(\chi )$, respectively), and the coupling strength between the mediator and the DM particle, $\lambda _b$ [22]. For each pair of mass values considered, $\lambda _b$ is set to the value, generally larger than one, predicting a DM relic density compatible with the astrophysical observations as detailed in Ref. [22]. Strong-interaction pair production of $\phi _b$, which does not depend on the coupling, is equivalent to the pair production of the lightest supersymmetric partner of the bottom quark (bottom squark, $\tilde{b}_1$) assuming that it decays exclusively into a bottom quark and the lightest neutralino ($\tilde{\chi }^0_1$). Exclusion limits on $m(\tilde{b}_1)$, which depend on $m(\tilde{\chi }^0_1)$, are set in dedicated searches by the ATLAS and CMS collaborations [25, 26]. The target of this search is the single production mode represented in Fig. 1(c), which can dominate the production rate of the $\phi _b$ mediator due to the relatively large values assumed for $\lambda _b$. The parameter space considered corresponds to $\phi _b$ masses of a few hundred GeV. A search by the ATLAS Collaboration with the $\sqrt{s} = 8\; \hbox {TeV}$ LHC Run-1 dataset has already excluded $m(\phi _b) < 600\;\hbox {GeV}$ for $m(\chi ) = 35\;\hbox {GeV}$ [27].

Four experimental signatures are considered in this paper. The first two signatures consist of event topologies with large missing transverse momentum and either one or two bottom quarks, while the other two consist of events with large missing transverse momentum and two top quarks, decaying either dileptonically or fully hadronically. The search presented in this paper is based on a set of independent analyses optimised for these four experimental signatures and searches for dark-matter production via colour-charged and colour-neutral mediators.

## Detector description and event reconstruction

The ATLAS experiment [28] is a multi-purpose particle detector with a forward-backward symmetric cylindrical geometry and nearly $4\pi$ coverage in solid angle.^2^ It consists of an inner tracking detector (ID) surrounded by a superconducting solenoid, electromagnetic and hadronic calorimeters, and an external muon spectrometer incorporating large superconducting toroidal magnets. The inner tracking detector consists of pixel and silicon microstrip detectors covering the pseudorapidity region $|\eta |<2.5$, surrounded by a transition radiation tracker which provides electron identification in the region $|\eta |<2.0$. Between Run 1 and Run 2, a new inner pixel layer, the insertable B-layer [29, 30], was inserted at a mean sensor radius of 3.3 cm. The inner detector is surrounded by a thin superconducting solenoid providing an axial 2 T magnetic field and by a fine-granularity lead/liquid-argon (LAr) electromagnetic calorimeter covering $|\eta |<3.2$. A steel/scintillator-tile calorimeter provides hadronic coverage in the central pseudorapidity range ($|\eta |<1.7$). The end-cap and forward regions ($1.5<|\eta |<4.9$) of the hadronic calorimeter are made of LAr active layers with either copper or tungsten as the absorber material. A muon spectrometer with an air-core toroid magnet system surrounds the calorimeters. Three stations of high-precision tracking chambers provide coverage in the range $|\eta |<2.7$, while dedicated chambers allow triggering in the region $|\eta |<2.4$. The ATLAS trigger system consists of a hardware-based level-1 trigger followed by a software-based high-level trigger [31].

The events used in this analysis are required to pass either an online trigger requiring a minimum of two electrons, two muons or an electron and a muon, or an online missing transverse momentum trigger selection. The trigger thresholds are such that a plateau of the efficiency is reached for events passing the analysis requirements presented in Sect. 4. The events are also required to have a reconstructed vertex [32] with at least two associated tracks with transverse momentum ($p_{\text {T}}$) larger than $400\;\hbox {MeV}$ which are consistent with originating from the beam collision region. The vertex with the highest scalar sum of the squared transverse momenta of the associated tracks is considered to be the primary vertex of the event.

This analysis requires the reconstruction of jets, muons, electrons, photons and missing transverse momentum. Jets are reconstructed from three-dimensional energy clusters in the calorimeter [33] using the anti-$k_t$ jet clustering algorithm [34] with a radius parameter $R=0.4$ implemented in the FastJet package [35]. Jets are calibrated as described in Ref. [36], and the expected average energy contribution from clusters resulting from additional *pp* interactions in the same or nearby bunch crossings (pile-up interactions) is subtracted according to the jet area [37]. Only jet candidates (baseline jets) with $p_{\text {T}} >20\;\hbox {GeV}$ and $|\eta |<2.8$ are considered in the analysis. Quality criteria identify jets arising from non-collision sources or detector noise and any event containing such a jet is removed [38, 39]. Additional selection requirements are imposed on jets with $p_{\text {T}} < 60\;\hbox {GeV}$ and $|\eta |<2.4$ in order to reject jets produced in pile-up interactions [40]. Jets are also reclustered into larger-radius jets ($R=0.8$ or 1.2) by applying the anti-$k_t$ clustering algorithm to the $R=0.4$ jets. These jets are exploited to identify *W*-boson decays into a pair of quarks and also to identify top-quark candidates.

Jets containing *b*-hadrons ($b\text {-jets}$) and which are within the inner detector acceptance ($|\eta |<2.5$) are identified ($b\text {-tagged}$) with a multivariate algorithm that exploits the impact parameters of the charged-particle tracks, the presence of secondary vertices and the reconstructed flight paths of *b*- and *c*-hadrons inside the jet [41, 42]. Depending on the signal region requirements detailed in Sect. 4, a “medium” or “tight” working-point is used for the $b\text {-jet}$ identification, corresponding to an average efficiency for *b*-quark jets in simulated $t\bar{t}$ events of 77 and 60%, respectively. An additional “loose” working-point with 85% efficiency for *b*-quark jets in simulated $t\bar{t}$ events is used to resolve ambiguities in the reconstruction of physics objects, as described at the end of this section.

Muon candidates are reconstructed in the region $|\eta |<2.7$ from muon spectrometer tracks matching ID tracks (where applicable). The pseudorapidity requirements are restricted to $|\eta |<2.4$ for events passing the muon online trigger criteria, due to the coverage of the muon triggering system. Events containing one or more muon candidates that have a transverse (longitudinal) impact parameter with respect to the primary vertex larger than 0.2 mm (1 mm) are rejected to suppress muons from cosmic rays. Baseline candidate muons, used for the definition of vetoes in all signal regions but those searching for fully hadronic top decays, must have $p_{\text {T}} >{10}{\hbox {GeV}}$ and pass the “medium” identification requirements defined in Ref. [43]. The baseline candidate muons used in fully hadronic $t\bar{t}$ final states are instead required to pass the “loose” identification requirements [43] and to have $p_{\text {T}} >{6}{\hbox {GeV}}$, in order to strengthen the veto definition. Baseline electron candidates are reconstructed from isolated electromagnetic calorimeter energy deposits matched to ID tracks and are required to have $|\eta |<2.47$ and $p_{\text {T}} >{10}{\hbox {GeV}}$, and must pass a “loose” likelihood-based identification requirement [44, 45].

Stricter requirements are imposed on the baseline lepton (electron or muon) definitions for the selection criteria requiring leptons in the final state. Signal muon candidates, used for all selection requirements with leptons in the final state, must have $p_{\text {T}} > 20\;\hbox {GeV}$ and satisfy “medium” identification criteria [43]. Furthermore, they are required to be isolated using a “loose” criterion designed to be 99% efficient for muons from *Z*-boson decays [43]. Signal electron candidates are required to pass “tight” requirements on the likelihood-based identification [44] and must have $p_{\text {T}} > 20\;\hbox {GeV}$. In order to improve signal acceptance, the requirement on the likelihood-based identification is relaxed to “medium” for the signal region optimised for the two-lepton final state. Like the muons, signal electrons are required to be isolated from other activity using a “loose” isolation criterion [46]. Signal electrons (muons) are matched to the primary vertex (PV) of the event (see Sect. 4) by requiring their transverse impact parameter $d^{\mathrm P \mathrm V}_0$, with respect to the primary vertex, to have a significance $|d^{\mathrm P \mathrm V}_0/\sigma (d^{\mathrm P \mathrm V}_0)| < 5\; (3)$. In addition, for both the electrons and muons the longitudinal impact parameter $z^{\mathrm P \mathrm V}_0$ and the polar angle $\theta$ are required to satisfy $|z^{\mathrm P \mathrm V}_0 \sin \theta | < 0.5$ mm. In the following, the combination of signal electrons and muons optimised for the two-lepton final state is referred to as the medium-lepton requirement. Similarily, the combination of the signal electrons and muons passing the “tight” identification criteria is referred to as the tight-lepton requirement. The number of leptons passing the medium and tight requirements is denoted by $\mathcal {N}^{\mathrm M}_{\ell }$ and $\mathcal {N}^{\mathrm T}_{\ell }$, respectively.

Photons are reconstructed from clusters of energy deposits in the electromagnetic calorimeter measured in projective towers [47, 48]. Photon candidates are required to have $p_{\text {T}} > 10\; \hbox {GeV}$ and $|\eta |<2.37$, whilst being outside the transition region $1.37< |\eta | < 1.52$ between the barrel and end-cap calorimeters, and to satisfy “tight” identification criteria [48]. The photons used in this analysis are further required to have $p_{\text {T}} > 130\; \hbox {GeV}$ and to be isolated [47].

To resolve reconstruction ambiguities, an overlap removal algorithm is applied to loose candidate leptons and jets. Jet candidates with $p_{\text {T}} >20\;\hbox {GeV}$ and $|\eta |<2.8$ are removed if they are not *b*-tagged when employing the loose working-point and are within $\Delta R =\sqrt{(\Delta y)^2+(\Delta \phi )^2} = 0.2$ of an electron candidate. The same is done for jets which lie close to a muon candidate and have less than three associated tracks or a ratio of muon $p_{\text {T}}$ to jet $p_{\text {T}}$ greater than 0.5. Finally, any lepton candidate within $\Delta R = 0.4$ of the direction of a surviving jet candidate is removed, in order to reject leptons from the decay of a *b*- or *c*-hadron. Electrons which share an ID track with a muon candidate are also removed.

The missing transverse momentum vector, $\vec {p}_{\text {T}}^{\text {miss}}$, whose magnitude is denoted by $E_{\text {T}}^{\text {miss}}$, is defined as the negative vector sum of the transverse momenta of all identified physics objects (electrons, photons, muons, jets) and an additional soft term. The soft term is constructed from all tracks that originate from the primary vertex but are not associated with any physics object. In this way, the $E_{\text {T}}^{\text {miss}}$ is adjusted for the calibration of the jets and the other identified physics objects above, while maintaining pile-up independence in the soft term [49, 50].

## Data and simulated event samples

The dataset used in this analysis consists of *pp* collision data recorded at a centre-of-mass energy of $\sqrt{s} = 13~\hbox {TeV}$ with stable beam conditions. The integrated luminosity of the combined 2015 + 2016 dataset after requiring that all detector subsystems were operational during data recording is $36.1 \; \mathrm {fb}^{-1}$. The uncertainty in the total integrated luminosity is 3.2%, derived following a methodology similar to that detailed in Ref. [51].

Monte Carlo (MC) simulated event samples are used to aid in the estimation of the background from SM processes and to model the dark-matter signal. All simulated events were processed through an ATLAS detector simulation [52] based on Geant4 [53] or through a fast simulation using a parameterisation of the calorimeter response and Geant4 for the other parts of the detector [54]. The simulated events are reconstructed with the same reconstruction algorithms used for data. Correction factors are applied to the simulated events to compensate for differences between data and MC simulation in the *b*-tagging efficiencies and mis-tag rates, lepton and photon identification, reconstruction and trigger efficiencies. The MC samples are reweighted so that the pile-up distribution matches the one observed in the data.

The matrix element (ME) generator, parton shower (PS), cross-section normalisation, parton distribution function (PDF) set and the set of tuned parameters (known as tune) describing the underlying event for these samples are given in Table 1, and more details of the generator configurations can be found in Refs. [55–58]. The generation of $t\bar{t}$ pairs and single-top-quark processes in the *Wt*- and *s*-channels was performed using the Powheg-Box v2 generator with the CT10 PDF set for the matrix element calculations. Electroweak *t*-channel single-top-quark events were generated using the Powheg-Box v1 generator. For all processes, a top-quark mass of 172.5 GeV is assumed. The PS and the underlying event were simulated using Pythia 6.428 with the CT10 PDF set. Samples of single-top-quark and $t\bar{t}$ production are normalised to their NNLO cross-section including the resummation of soft gluon emission at next-to-next-to-leading-log (NNLL) accuracy using Top++2.0 [59–61].

Events containing *W* or *Z* bosons with associated jets, including jets from the hadronisation of *b*- and *c*-quarks, were simulated using the Sherpa v2.2.1 generator. Matrix elements were calculated for up to two additional partons at next-to-leading order (NLO) and four partons at leading order (LO) using the Comix [62] and Open Loops [63] matrix element generators and merged with the Sherpa PS [64] using the ME+PS@NLO prescription [65]. The NNPDF30NNLO [66] PDF set was used in conjunction with the dedicated PS tune developed by the Sherpa authors.

Diboson and triboson processes were also simulated using the Sherpa generator using the NNPDF30NNLO PDF set in conjunction with a dedicated PS tune developed by the Sherpa authors. Matrix elements for these samples were calculated for up to one (diboson processes) or zero (triboson processes) additional partons at NLO and up to three (diboson processes) or two (triboson processes) additional partons at LO. Additional contributions to the SM backgrounds in the signal regions arise from the production of $t\bar{t}$ pairs in association with *W*/*Z*/*h* bosons and possibly additional jets. These processes were modelled by event samples generated at NLO using the MadGraph5_aMC NLO [67] v2.2.3 generator and showered with the Pythia v8.186 PS.

**Table 1:** Simulated signal and background event samples: the corresponding generator, parton shower, cross-section normalisation, PDF set and underlying-event tune are shown

Physics process	Generator	Parton shower	Cross-section normalisation	PDF set	Tune
Dark-matter signals	MadGraph 2.3.3 [67]	Pythia 8.212 [68]	NLO [69, 70]	NNPDF23LO [71]	A14 [72]
$W(\rightarrow \ell \nu )$ + jets	Sherpa 2.2.1 [73]	Sherpa 2.2.1	NNLO [74]	NNPDF30NNLO [71]	Sherpa default
$Z/\gamma ^{*}(\rightarrow \ell \ell )$ + jets	Sherpa 2.2.1	Sherpa 2.2.1	NNLO [74]	NNPDF30NNLO	Sherpa default
$t\bar{t}$	powheg-box v2 [75]	Pythia 6.428 [76]	NNLO+NNLL [77–82]	NLO CT10 [71]	Perugia2012 [83]
Single-top					
(*t*-channel)	powheg-box v1	Pythia 6.428	NNLO+NNLL [59]	NLO CT104f	Perugia2012
Single-top					
(*s*- and *Wt*-channel)	powheg-box v2	Pythia 6.428	NNLO+NNLL [60, 61]	NLO CT10	Perugia2012
$t\bar{t}+W/Z/\gamma ^{*}/h$	MadGraph5_aMC@NLO 2.2.3 (NLO)	Pythia 8.186	NLO [67]	NNPDF30NLO	A14
Diboson	Sherpa 2.2.1 [73]	Sherpa 2.2.1	NLO	NNPDF30NNLO	Sherpa default
$h+W/Z$	MadGraph5_aMC@NLO 2.2.3 (NLO)	Pythia 8.186	NLO [84]	NNPDF30NLO	A14
$t\bar{t}+WW/t\bar{t}$	MadGraph5_aMC@NLO 2.2.3 (LO)	Pythia 8.186	NLO [67]	NNPDF23LO	A14
$t+Z/WZ/t\bar{t}$	MadGraph5_aMC@NLO 2.2.3 (LO)	Pythia 8.186	LO	NNPDF23LO	A14
Triboson	Sherpa 2.2.1	Sherpa 2.2.1	NLO	NNPDF30NNLO	Sherpa default

In all MC samples, except those produced by Sherpa, the EvtGen v1.2.0 program [85] was used to model the properties of the bottom and charm hadron decays. All Pythia v6.428 samples used the PERUGIA2012 [83] tune for the underlying event, while Pythia v8.186 and Herwig++ showering were run with the A14 and UEEE5 [86] underlying-event tunes, respectively. To simulate the effects of additional *pp* collisions in the same and nearby bunch crossings, additional interactions were generated using the soft QCD processes of Pythia 8.186 with the A2 tune [87] and the MSTW2008LO PDF [88], and overlaid onto each simulated hard-scatter event.

Alternative samples are employed to derive systematic uncertainties associated with the specific configuration of the MC generators used for the nominal SM background samples, as detailed in Sect. 6. They include variations of the renormalisation and factorisation scales, the CKKW-L matching [89] scale, as well as different PDF sets and hadronisation models.

The event generation for the dark-matter signal samples followed the prescriptions in Ref. [13]. Events were generated from leading-order (LO) matrix elements using the MadGraph generator v2.3.3 interfaced to Pythia v8.212 with the A14 tune for the modelling of the top-quark decay chain (when applicable), parton showering, hadronisation and the description of the underlying event. The renormalisation and factorisation scale choice adopted is the default MadGraph dynamical scale as documented in Ref. [90]. For the $b\bar{b}$ +$\phi /a$ and $t\bar{t}$ +$\phi /a$ models the events were generated with up to one additional parton, while for the $b$-FDM models the events were generated with up to two additional partons. The $t\bar{t}$ +$\phi /a$ and $b$-FDM samples were generated in the 5-flavour scheme, while the $b\bar{b}$ +$\phi /a$ samples were generated in the 4-flavour scheme. Following Ref. [13], the minimum $p_{\text {T}}$ requirement for $b\text {-jets}$ in the final state in MadGraph was set to $30\;\hbox {GeV}$ for the $b\bar{b}$ +$\phi /a$ model, in order to increase the number of events in the relevant phase space for the analysis. This requirement does not affect the MC signal sample passing the event selection. The PDF set NNPDF23LO was used, adopting $\alpha _{\text {S}} = 0.130$ and either the 5-flavour or the 4-flavour scheme consistently with the choice made for generating the events. The jet–parton matching was realised following the CKKW-L prescription. For the $t\bar{t}$ +$\phi /a$ model the matching scale was set to one quarter of the mass of the particle mediating the interaction between the SM and DM sectors. For the $b\bar{b}$ +$\phi /a$ and $b$-FDM models the matching scale was set to 30 GeV. The coupling *g* between the colour-neutral mediator for the $t\bar{t}$ +$\phi /a$ and $b\bar{b}$ +$\phi /a$ models and both the SM and the dark sector was assumed to be one, which implies pure Yukawa-type couplings between the mediator and the SM quarks. This choice impacts the mediator width and cross-section calculation for these models, but it was shown to have no significant impact on the kinematic properties [13].

For the $t\bar{t}$ +$\phi /a$ and $b\bar{b}$ +$\phi /a$ models the production cross-section was computed at NLO accuracy in the strong coupling constant $\alpha _{\text {S}}$ using the MadGraph5_aMC@NLO generator with the NNPDF30NLO PDF set using $\alpha _{\text {S}} = 0.118$. For this procedure a dynamical scale equal to $P_\mathrm {T}/2$ was adopted, with $P_\mathrm {T}$ being the scalar sum of the transverse momenta of all final-state particles. The flavour scheme adopted is consistent with that used for event generation. For the mass range in which this analysis is sensitive, the NLO value of the cross-sections for the $t\bar{t}$ +$\phi /a$ model is about 25% larger than the corresponding LO value [69, 70]. For the $b\bar{b}$ +$\phi /a$ samples the NLO value of the cross-section is between 56% and 75% of the corresponding LO value. This is driven by the MadGraph minimum $b\text {-jet}$ $p_{\text {T}}$ requirement due to the strong dependence of the NLO cross-section on this parameter. For the $b$-FDM signal models, the cross-section was computed at LO accuracy using the MadGraph5_aMC@NLO generator and the same flavour scheme used for the event generation.

## Event selection

Five signal regions (SR) are defined and optimised to detect dark-matter production via spin-0 mediators. Two signal regions, SRb1 and SRb2, are optimised for models in which dark matter is produced in conjunction with one or two *b*-quarks, respectively. Specifically, SRb1 is designed to optimally select candidate signal events of the colour-charged scalar mediator models (bFDM) introduced in Sect. 1. SRb2 focuses instead on scalar and pseudoscalar colour-neutral mediators and was specifically optimised for low mediator masses (below $200\;\hbox {GeV}$). These SRs require events with no leptons and low jet multiplicity. SRt1, SRt2 and SRt3 are optimised to detect events in which DM is produced in association with a $t\bar{t}$ pair, which either decays fully hadronically (SRt1 and SRt2) or dileptonically (SRt3). The SRt1 and SRt2 SRs are optimised for low ($< 100\;\hbox {GeV}$) and high (between 100 and $350\;\hbox {GeV}$) mediator mass assumptions, respectively, and are assigned fully hadronic events with high jet multiplicity. The regions SRt1 and SRt2 overlap in terms of their selection criteria. The region SRt3 focuses on mediator masses below $100\;\hbox {GeV}$ and contains events with two leptons in the final state.

### Signatures with *b*-quarks and $E_{\text {T}}^{\text {miss}}$

Events assigned to SRb1 and SRb2 are required to pass the missing transverse momentum trigger and to have at least one jet ($\mathcal {N}_j$). A minimum azimuthal angle between the directions of the missing transverse momentum and any of the jets in the event ($\Delta \phi (\mathrm {j}, \vec {p}_{\text {T}}^{\text {miss}})$) is required, in order to reduce the contamination by multi-jet events where fake $E_{\text {T}}^{\text {miss}}$ arises from jet energy mismeasurements or semileptonic decays of hadrons inside jets. Events with at least one baseline muon or electron ($\mathcal {N}^{\mathrm B}_{\ell }$) are discarded to reject leptonic decays of *W* and *Z* bosons. The dominant background processes for the events passing these requirements are $t\bar{t}$ and $Z \text {\,+\,jets}$ processes.

Events with at least one tight $b\text {-tagged}$ jet ($\mathcal {N}^{\mathrm T}_{b}$) and which pass the kinematic requirements specified in Table 2 are assigned to SRb1. The high-$E_{\text {T}}^{\text {miss}}$ selection required is essential to discriminate the signal from the background in this SR. An upper limit on the scalar sum of the transverse momenta of the baseline jets in the events excluding the leading and subleading jets ($H_\mathrm {T3}$ [25]) is used in this SR to reduce the contributions from top-quark pair-production processes.

Events assigned to SRb2 have instead at least two tight $b\text {-tagged}$ jets. When the $b\text {-tagged}$ jet multiplicity is different from two, the $b\text {-tagged}$ jets are sorted in descending order according to their *b*-tagging probability. For this SR, a requirement of low jet multiplicity was found to be more effective in reducing the $t\bar{t}$ background. The jet multiplicity of candidate signal events is required to not exceed three, and the transverse momentum of the third jet in the event must not exceed $60\;\hbox {GeV}$. For the same purpose, the ratio of the transverse momentum of the leading jet to $H_\mathrm {T}$, the scalar sum of the transverse momenta of all jets in the events, ($H_\mathrm {T}^\mathrm {ratio}= p_{\text {T}} (j_1) / H_\mathrm {T}$) is required to be larger than 75%.

**Table 2:** Summary of the kinematic and topology-dependent selections for signal regions SRb1 and SRb2

Observable	SRb1	SRb2
Trigger	$E_{\text {T}}^{\text {miss}}$	
$\mathcal {N}_{j}$	$\ge 2$	2 or 3
$\mathcal {N}^{\mathrm T}_{b}$	$\ge 1$	$\ge 2$
$\mathcal {N}^{\mathrm B}_{\ell }$	0	
$E_{\text {T}}^{\text {miss}}$ [GeV]	$>650$	$>180$
$p_{\text {T}} (bj_1)$ [GeV]	$>160$	$>150$
$p_{\text {T}} (j_1)$ [GeV]	$>160$	$>150$
$p_{\text {T}} (j_2)$ [GeV]	$>160$	$>20$
$p_{\text {T}} (j_3)$ [GeV]	–	$<60$
$$H_\mathrm {T3}$$ [GeV]	$$< 100 $$	–
$H_\mathrm {T}^\mathrm {ratio}$	–	$>0.75$
$\delta ^-$ [rad]	–	$< 0$
$\delta ^+$ [rad]	–	$<0.5$
Multi-jet rejection specific		
$\Delta \phi (\mathrm {j}, \vec {p}_{\text {T}}^{\text {miss}})$ [rad]	$>0.6$	$>0.4$

The azimuthal separations between the $b\text {-tagged}$ jets ($\Delta \phi _{bb}$) and the $\Delta \phi (\mathrm {j}, \vec {p}_{\text {T}}^{\text {miss}})$ are exploited to enhance the separation between the signal and the irreducible background in this channel ($Z(\nu \bar{\nu })$+$b\bar{b}$), as the latter is characterised by small $\Delta \phi _{bb}$ values when the $b\text {-jets}$ originate from the gluon-splitting process. Linear combinations of these two variables are used to define the selection criteria in Table 2:

$$\begin{aligned} \delta ^-&= \Delta \phi (\mathrm {j}, \vec {p}_{\text {T}}^{\text {miss}})- \Delta \phi _{bb}, \\ \delta ^+&= \vert \Delta \phi (\mathrm {j}, \vec {p}_{\text {T}}^{\text {miss}})+ \Delta \phi _{bb}- \pi \vert . \end{aligned}$$

An additional handle to discriminate between the $b\bar{b}+\phi$ and $b\bar{b}+a$ signal models and the background is the spin of the particle decaying into invisible decay products. It was shown in Ref. [91] that it is possible to discriminate between such scalar, pseudoscalar and vector particles by exploiting information about the production angle of the visible particles with respect to the proton beam axis. A convenient variable to exploit this feature, proposed in Ref. [92] relies on the pseudorapidity difference between the two $b\text {-tagged}$ jets ($\Delta \eta _{bb}$):

$$\begin{aligned} \cos {\theta }^*_{bb}= \left| \tanh {\left( \frac{\Delta \eta _{bb}}{2}\right) }\right| . \end{aligned}$$

The variable $\cos {\theta }^*_{bb}$, evaluated in the laboratory frame, is the key observable used in SRb2 to discriminate the signal from the background. The distribution of $\cos {\theta }^*_{bb}$ is approximately flat for $b\text {-jets}$ produced in association with scalar or vector particles with masses below $100\;\hbox {GeV}$, while it exhibits a pronounced enhancement at values near one for pseudoscalar particles in the same mass range. In order to further enhance the sensitivity to the signal, the signal region SRb2 is divided into four independent bins in $\cos {\theta }^*_{bb}$: SRb2-bin1 (0, 0.25), SRb2-bin2 (0.25, 0.5), SRb2-bin3 (0.5, 0.75), SRb2-bin4 (0.75, 1.0), which are statistically combined in the final result.

### Signatures with top quarks and $E_{\text {T}}^{\text {miss}}$

**Table 3:** Summary of the kinematic and topology-dependent selections for signal regions SRt1, SRt2 and SRt3

Observable	SRt1	SRt2	SRt3
Trigger	$E_{\text {T}}^{\text {miss}}$		$2\ell$
$\mathcal {N}_{j}$	$\ge 4$		$\ge 1$
$\mathcal {N}^{\mathrm M}_{b}$	$\ge 2$		$\ge 1$
$\mathcal {N}^{\mathrm B}_{\ell }$	0		–
$\mathcal {N}^{\mathrm M}_{\ell }$	–		2 OS
$\mathcal {N}_{\tau }$	0		–
$E_{\text {T}}^{\text {miss}}$ [GeV]	$>300$		–
$p_{\text {T}} (bj_1)$ [GeV]	$>20$		$>30$
$p_{\text {T}} (j_1, j_2)$ [GeV]	$>80, 80$		$>30$
$p_{\text {T}} (j_3, j_4)$ [GeV]	$>40, 40$		–
$p_{\text {T}} (\ell _1, \ell _2)$ [GeV]	–		$>25, 20$
$$m_{\ell \ell }$$ [GeV]	–		$$ > 20$$
$$\vert m_{\ell \ell }^{\mathrm {SF}} - m_Z \vert $$ [GeV]	–		$$ > 20$$
$m^{\mathrm {jet\;1,2}}_{\mathrm {R=0.8}}$ [GeV]	$>80,80$	–	–
$m^{\mathrm {jet\;1,2}}_{\mathrm {R=1.2}}$ [GeV]	–	$>140,80$	–
$m_{\mathrm {T}}^{b,\mathrm {min}}$ [GeV]	$>150$	$>200$	–
$m_{\mathrm {T}}^{b,\mathrm {max}}$ [GeV]	$>250$	–	–
$\Delta R_{bb}$	$>1.5$	$>1.5$	–
$E_{\text {T}}^{\text {miss, sig}}$ [$\sqrt{\hbox {GeV}}$]	–	$>12$	–
$\Delta \phi _{\mathrm {boost}}$ [rad]	–		$< 0.8$
$m^{\mathrm {min}}_{b2\ell }$ [GeV]	–		$<170$
$\xi ^+$ [GeV]	–		$>170$
$m_{\mathrm {T2}}^{\ell \ell }$ [GeV]	–		$>100$
Multi-jet rejection specific			
$\Delta \phi (\mathrm {j}, \vec {p}_{\text {T}}^{\text {miss}})$ [rad]	$>0.4$		–
$E_{\text {T}}^{\text {miss,track}}$ [GeV]	$> 30$		–
$\Delta \phi (\vec {p}_{\text {T}}^{\text {miss}},\vec {p}_{\text {T}}^{\text {miss,track}})$ [rad]	$< \pi /3$		–

Events assigned to SRt1 and SRt2 are required to contain at least four jets. At least two jets in every event must be $b\text {-tagged}$ at the medium working-point ($\mathcal {N}^{\mathrm M}_{b}$). Events containing baseline electrons and muons are discarded. Furthermore, events with a $\tau$-candidate are also rejected ($\mathcal {N}_\tau = 0$). The $\tau$-candidate is defined as a jet with less than four associated tracks which has not passed the medium *b*-tagging requirement and which has a $\phi$ separation from the $\vec {p}_{\text {T}}^{\text {miss}}$ of no more than $\pi /5$ radians. Events are required to pass the missing transverse momentum trigger and to satisfy $E_{\text {T}}^{\text {miss}} > 300\; \hbox {GeV}$. Also in this SRs, a minimum $\Delta \phi (\mathrm {j}, \vec {p}_{\text {T}}^{\text {miss}})$ requirement is applied in order to reject events with $E_{\text {T}}^{\text {miss}}$ arising from mismeasurements and semileptonic decays of hadrons inside jets. Further rejection of such events is achieved by additional requirements on the missing transverse momentum computed using only the information from the tracking system ($\vec {p}_{\text {T}}^{\text {miss,track}}$, with magnitude $E_{\text {T}}^{\text {miss,track}}$) and its angle with respect to the $\vec {p}_{\text {T}}^{\text {miss}}$ ($\Delta \phi (\vec {p}_{\text {T}}^{\text {miss}},\vec {p}_{\text {T}}^{\text {miss,track}})$). The dominant backgrounds for these signal regions are top-quark pair production, *Z*+jets, and the production of a *Z* boson in association with $t\bar{t}$. Four main observables are exploited to discriminate DM signal events from the SM background processes: $m_{\mathrm {T}}^{b,\mathrm {min}}$, $m_{\mathrm {T}}^{b,\mathrm {max}}$, $E_{\text {T}}^{\text {miss, sig}}$, and $\Delta R_{bb}$. The variables $m_{\mathrm {T}}^{b,\mathrm {min}}$ and $m_{\mathrm {T}}^{b,\mathrm {max}}$ are defined as the transverse mass^3^ of the $\vec {p}_{\text {T}}^{\text {miss}}$ vector and $b\text {-tagged}$ jet with the smallest and largest angular distance^4^ from it, respectively. The $m_{\mathrm {T}}^{b,\mathrm {min}}$ variable is designed to be bounded from above by the top-quark mass for semileptonic $t\bar{t}$ decays, because the closest $b\text {-tagged}$ jet to the $\vec {p}_{\text {T}}^{\text {miss}}$ vector usually belongs to the leg of the decay where the $W$ boson decays into leptons. The variable $m_{\mathrm {T}}^{b,\mathrm {max}}$ recovers the discriminating power in the case of wrong pairing. The $E_{\text {T}}^{\text {miss, sig}}$ variable is defined as the ratio of the $E_{\text {T}}^{\text {miss}}$ to the square-root of the scalar sum of the transverse momenta of all jets in the events ($H_\mathrm {T}$) to discriminate the high-mediator-mass signal models in SRt2 from the SM background. Finally, the angular distance between the two $b\text {-tagged}$ jets in the event ($\Delta R_{bb}$) is exploited to suppress $Z(\nu \nu )$+$b\bar{b}$ events where the two *b*-quarks arise from gluon-splitting and are characterised by a small angular separation.

The SRt1 selection is optimised for low-mass spin-0 mediators ($m(\phi /a) < 100\;\hbox {GeV}$). Requirements on the two leading reclustered jet masses with radius 0.8 ($m^{\mathrm {jet\;1}}_{\mathrm {R=0.8}}$, $m^{\mathrm {jet\;2}}_{\mathrm {R=0.8}}$) exploit the presence of boosted hadronic decays of $W$ bosons from top quarks in the event. The requirements applied in SRt1 are such that both reclustered jets are compatible with a *W*-boson candidate. The SRt2 signal region is optimised instead for high-mass spin-0 mediators ($100\;\hbox {GeV}< m(\phi /a) < 350\;\hbox {GeV}$). Requirements on the two leading reclustered jet masses with radius 1.2 ($m^{\mathrm {jet\;1}}_{\mathrm {R=1.2}}$, $m^{\mathrm {jet\;2}}_{\mathrm {R=1.2}}$) are used to exploit the more boosted topology of these signal events compared to the backgrounds. The requirements applied in SRt2 are such that the leading large-radius jet is compatible with a top-quark candidate and the subleading large-radius jet is compatible with a *W*-boson candidate. The specific requirements for each discriminating observable in SRt1 and SRt2 are summarised in Table 3.

Finally, events assigned to SRt3 are required to have exactly two opposite-sign leptons ($\mathcal {N}^{\mathrm M}_{\ell } = 2$ OS), electrons or muons, either same- or different-flavour, with an invariant mass (regardless of the flavours of the leptons in the pair), $m_{\ell \ell }$, being larger than $20\;\hbox {GeV}$. In addition, for same-flavour lepton pairs, events with $m_{\ell \ell }$ within $20\; \hbox {GeV}$ of the $Z$-boson mass are vetoed. Furthermore, candidate signal events are required to have at least one medium $b\text {-tagged}$ jet. Events are required to pass the two-lepton triggers and the leading and subleading lepton transverse momenta in the event are required to be at least 25 and $20 \;\hbox {GeV}$, respectively, which also guarantees that the plateau of efficiency of the triggers is reached. The main reducible backgrounds for this analysis are dileptonic $t\bar{t}$ decays, $Z \text {\,+\,jets}$ and dibosons. The main handle for the rejection of these backgrounds is the lepton-based “stransverse mass”, $m_{\mathrm {T2}}^{\ell \ell }$ [93–95], which is a kinematic variable with an endpoint at the *W*-boson mass for events containing two $W$ bosons decaying into leptons. In this selection it is used in linear combination with the $E_{\text {T}}^{\text {miss}}$, in order to maximise the discrimination power of the two variables [91]:

$$\begin{aligned} \xi ^+= m_{\mathrm {T2}}^{\ell \ell }+ 0.2 \cdot E_{\text {T}}^{\text {miss}}. \end{aligned}$$

Further requirements are placed on $\Delta \phi _{\mathrm {boost}}$ [93], the azimuthal angular distance between $\vec {p}_{\text {T}}^{\text {miss}}$ and the vector sum of $\vec {p}_{\text {T}}^{\text {miss}}$ and the transverse momentum of the leptons, and on $m^{\mathrm {min}}_{b2\ell }$, which is the smallest invariant mass computed between the $b\text {-tagged}$ jet and each of the two leptons in the event. Both variables are used to further reject residual contamination from reducible backgrounds for this selection. The variable $\Delta \phi _{\mathrm {boost}}$, can be interpreted as the azimuthal angular difference between the $\vec {p}_{\text {T}}^{\text {miss}}$ and the opposite of the vector sum of all the transverse hadronic activity in the event. The requirement on this variable reject $Z(\ell ^+\ell ^-)$+jets events where the $E_{\text {T}}^{\text {miss}}$ arises from jet mismeasurements, while retaining a large fraction of the signal. In events with two top quarks decaying dileptonically such as in the signal topology, at least one of the two mass combinations must be bounded from above by $m^{\mathrm {min}}_{b2\ell }< \sqrt{m_t^2 - m_W^2}$. This variable helps to reject residual reducible backgrounds, while retaining 99% of the signal. The specific requirements for SRt3 are summarised in Table 3.

## Background estimation

The SM backgrounds contributing to each of the five SRs are estimated with the aid of the MC simulation and using control regions (CRs) constructed to enhance a particular background and to be kinematically similar but orthogonal to the SRs. The expected background is determined separately in each SR through a profile likelihood fit based on the HistFitter package [96]. The CR yields constrain the normalisation of the dominant SM background processes. Such normalisation factors are treated as free fit parameters and are uncorrelated between fits of different SRs. The systematic uncertainties are included as nuisance parameters in the fit. In the case of a “background-only” fit set-up, only the CRs are considered and the signal contribution is neglected. The number of background events predicted by simulation in the SRs is normalised according to the results of the fit. When computing exclusion limits as described in Sect. 7, the SRs are also used to constrain the background predictions. The non-dominant SM backgrounds are determined purely from MC simulation, except fake or non-prompt lepton backgrounds (arising from jets misidentified as leptons or produced in either hadron decays or photon conversions) and the multi-jet background, both of which are estimated using a data-driven method described below. The background estimates in the SRs are validated by extrapolating the results of the likelihood fit in the CRs to dedicated validation regions (VRs), which are designed to be orthogonal to both the signal and control regions. In all CRs and VRs used in this analysis the signal contamination was found to be negligible.

An important source of background for all 0-lepton signal regions is *Z* bosons decaying into neutrinos when produced in conjunction with one or more jets emanating from heavy-flavour quarks. Production of top-quark pairs is a substantial background source for all selections except for SRb1, where the very high $E_{\text {T}}^{\text {miss}}$ requirement rejects this background. More specifically, top-quark pairs with at least one of the *W* bosons decaying into leptons (where the lepton is either a non-identified electron or muon, or a hadronically decaying $\tau$ lepton) enter SRb2, SRt1 and SRt2, while events with both *W*-bosons decaying into leptons enter SRt3. Events from $t\bar{t} \text {+}Z$ production, when the *Z* boson decays into neutrinos, are an irreducible background for the three SRs targeting dark matter produced in association with top quarks.

The normalisation factor for the background arising from $Z\rightarrow \nu \bar{\nu }$ events is estimated from data in CRs with two tight same-flavour opposite-sign (SFOS) leptons ($\ell = (e, \mu )$) and an invariant mass compatible with the *Z*-boson mass. For these CRs, labelled in the following as CRZt1, CRZt2, CRZb1 and CRZb2, the $p_{\text {T}}$ of the leptons is added vectorially to the $\vec {p}_{\text {T}}^{\text {miss}}$ to mimic the expected missing transverse momentum spectrum of $Z\rightarrow \nu \bar{\nu }$ events, and is denoted in the following by $E_{\mathrm {T,\ell \ell }}^{\mathrm {miss}}$. Observables that make use of $E_{\text {T}}^{\text {miss}}$ in their definition are recalculated for these regions by using $E_{\mathrm {T,\ell \ell }}^{\mathrm {miss}}$ instead. These variables are $\delta ^-_{\ell \ell }$, $\delta ^+_{\ell \ell }$, $\Delta \phi (\mathrm {j}, \vec {p}_{\mathrm {T,\ell \ell }}^{\text {miss}})$, $m_{\mathrm {T,\ell \ell }}^{b,\mathrm {min}}$, $m_{\mathrm {T,\ell \ell }}^{b,\mathrm {max}}$ and $E_{\mathrm {T,\ell \ell }}^{\text {miss, sig}}$.

Single tight-lepton CRs, denoted by CRTb2, CRTt1 and CRTt2, are used to estimate the background from top-quark pairs in SRb2, SRt1 and SRt2. The transverse mass^5^ ($m_\mathrm {T}$) of the lepton and the $\vec {p}_{\text {T}}^{\text {miss}}$, and the angular distance between the lepton and the $b\text {-tagged}$ jet closest to it ($\Delta R^{\mathrm {min}}_{b\ell }$) are used to enhance the purity of top-quark events. In CRTt1 and CRTt2 the lepton is treated as a jet, in order to better mimic the type of background events that contaminate the corresponding SR. The dileptonic top background, which contaminates SRt3, is instead estimated in a two-medium-leptons CR composed of events that fail the $\xi ^+$ requirement (CRTt3).

Finally, $t\bar{t}$ +$V$ events, and in particular $t\bar{t} \text {+}Z$ events where the *Z* boson decays into neutrinos, represent the irreducible background for the three SRs targeting dark matter produced in association with top quarks. This background is estimated from data using two CRs. To estimate the normalisation factor for the $t\bar{t} \text {+}Z$ background in SRt1 and SRt2 a control region of $t\bar{t}$ +$\gamma$ events (CR$\gamma$) is used. Events with $p_{{\mathrm {T}}\gamma } > m_{\mathrm Z}$ are selected, for which the kinematic properties resemble those of $t\bar{t} \text {+}Z (\nu \nu )$. The CR$\gamma$ contains events with exactly one energetic tight photon ($\mathcal {N}_\gamma = 1$) and at least one lepton from the decay of the $t\bar{t}$ system. This strategy substantially increases the number of events at large missing transverse momentum and allows CR$\gamma$ to better mimic the hard kinematic requirements of SRt1 and SRt2. Furthermore, the $p_{\text {T}}$ of the photon is added vectorially to the $\vec {p}_{\text {T}}^{\text {miss}}$ to mimic the expected missing transverse momentum spectrum of $Z\rightarrow \nu \bar{\nu }$ events. The variable obtained with this procedure is referred to as $E_{\mathrm {T,\gamma }}^{\mathrm {miss}}$ in the following.

**Table 4:** Summary of the control region selections. Only the topological requirements modified with respect to Tables 2 and 3 are indicated. The symbol $\mathcal {N}_{b}$ refers to either $\mathcal {N}^{\mathrm M}_{b}$ or $\mathcal {N}^{\mathrm T}_{b}$ in order to be consistent with the SR definition for each region

Observable	CRZb1	CRZb2	CRZt1	CRZt2	CRTb2	CRTt1	CRTt2	CRTt3	CR$\gamma$	CR$3\ell$
Trigger	1$\ell$	1$\ell$	1$\ell$		1$\ell$	$E_{\text {T}}^{\text {miss}}$		$2\ell$	$1\gamma$	$2\ell$
$\mathcal {N}_{j}$	$\ge 2$	$2-3$	$\ge 4$		$2-3$	$\ge 3$		$\ge 1$	$\ge 4$	$$\begin{array}{c} \ge 3 \\ \ge 2 \end{array}$$ or $$\begin{array}{c} \ge 4 \\ =1 \end{array}$$
$\mathcal {N}_{b}$	$\ge 1$	$\ge 2$	$\ge 2$		$\ge 2$	$\ge 2$		$\ge 1$	$\ge 2$	
$\mathcal {N}^{\mathrm T}_{\ell }$	$=2$ (SFOS)		$=2$ (SFOS)		$=1$	$=1$		–	$=1$	–
$\mathcal {N}^{\mathrm M}_{\ell }$	–	–	–		–	–		$=2$ (OS)	–	3 (1 SFOS)
$\mathcal {N}_{\tau }$	–	–	0		–	0		–	–	–
$\mathcal {N}_{\gamma }$	–	–	–		–	–		$=1$	–	–
$$E_{\text {T}}^{\text {miss}}$$ [GeV]	$$<120$$	$$<60$$	$$ < 50$$		$$>180$$	$$>250$$		–	–	–
$$E_{\mathrm {T,\ell \ell }}^{\mathrm {miss}}$$ [GeV]	$$> 300 $$	$$> 120$$	$$>160$$		–	–		–	–	$$>80$$
$p_{\text {T}} (\gamma )$ [GeV]	–	–	–		–	0		–	$>150$	–
$p_{\text {T}} (\ell _1),p_{\text {T}} (\ell _2)$ [GeV]	$>30,>25$	$>30,>25$	$>28,>28$		$>30,-$	$>28,-$		$>25,20$	$>28$	$>25,>20$
Multi-jet rejection specific	As SR		no		As SR				No	As SR
$m_\mathrm {T}$ [GeV]	–	–	–		$>30$	[30–100]		–	–	$>30$
$\Delta R^{\mathrm {min}}_{b\ell }$[rad]	–	–	–		–	$<1.0$	$<1.5$	–	–	–
$\vert m_{\ell \ell }- m_Z \vert$ [GeV]	$<20$	$<30$	$<5$		–	–		as SR	–	$<10$
$\Delta \phi (\mathrm {j}, \vec {p}_{\mathrm {T,\ell \ell }}^{\text {miss}})$ [rad]	$>0.6$	–	–		–	–		–	–	–
$H_\mathrm {T}^\mathrm {ratio}$	–	$>0$	–		as SR	–		–	–	–
$\delta ^-_{\ell \ell }$, $\delta ^+_{\ell \ell }$ [rad]	–	$<1, <0.5$	–		as SR	–		–	–	–
$m^{\mathrm {jet\;0}}_{\mathrm {R=SR}}$ [GeV]	–	–	$>60$	$>60$	–	$>60$	$>140$	–	–	–
$m^{\mathrm {jet\;1}}_{\mathrm {R=SR}}$ [GeV]	–	–	–		–	$>60$	$>80$	–	–	–
$m_{\mathrm {T}}^{b,\mathrm {min}}$ [GeV]	–	–	–		–	$>100$		–	–	–
$m_{\mathrm {T}}^{b,\mathrm {max}}$ [GeV]	–	–	–		–	–		–	–	–
$m_{\mathrm {T,\ell \ell }}^{b,\mathrm {min}}$ [GeV]	–	–	–	$>100$	–	–		–	–	–
$m_{\mathrm {T,\ell \ell }}^{b,\mathrm {max}}$ [GeV]	–	–	$>100$	–	–	–		–	–	–
$\Delta R_{bb}$	–	–	0		–	1.5		–	–	–
$E_{\mathrm {T,\ell \ell }}^{\text {miss, sig}}$ [$\sqrt{\hbox {GeV}}$]	–	–	–	$>6$	–	–		–	–	–
$\xi ^+$ [GeV]	–	–	–		–	–		$< 150$	–	–
$m^{\mathrm {min}}_{b2\ell }$ [GeV]	–	–	–		–	–		$< 170$	–	–
$\xi ^{+}_{\ell \ell }$ [GeV]	–	–	–		–	–		–	–	$>120$
$m^{\mathrm {min}}_{2b\ell }$ [GeV]	–	–	–		–	–		–	–	$<170$

**Fig. 2:**
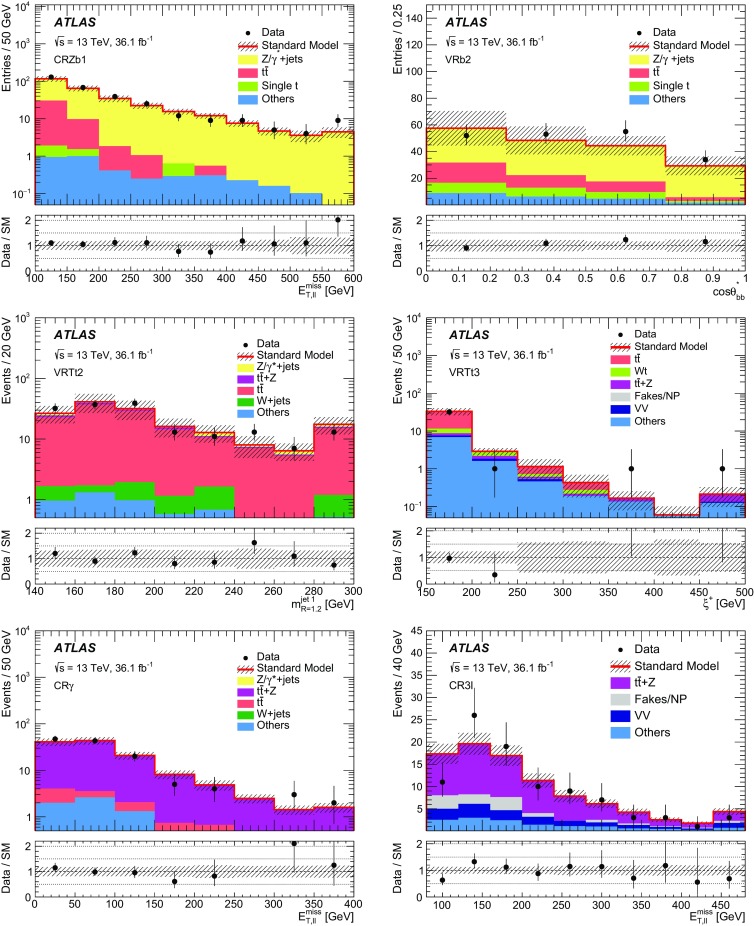
Comparison of the data with the post-fit Monte Carlo prediction of some kinematic distributions in control and validation regions. The bottom panel shows the ratio of the data to the Monte Carlo prediction. The band includes all systematic uncertainties defined in Sect. 6. The last bins include overflows, where applicable. The top left panel shows the $E_{\mathrm {T,\ell \ell }}^{\mathrm {miss}}$ distribution in CRZb1. The $E_{\mathrm {T,\ell \ell }}^{\mathrm {miss}}$ requirement is relaxed to $100\;\hbox {GeV}$. The other panels show the $\cos {\theta }^*_{bb}$ distribution in VRb2 (top right), the $m^{\mathrm {jet\;1}}_{\mathrm {R=1.2}}$ distribution in VRTt2 (middle left), the $\xi ^+$ distribution the VRTt3 (middle right), the $E_{\mathrm {T,\ell \ell }}^{\mathrm {miss}}$ distribution in CR$\gamma$ (bottom left) and the $E_{\mathrm {T,\ell \ell }}^{\mathrm {miss}}$ distribution in CR$3\ell$ (bottom right)

**Fig. 3:**
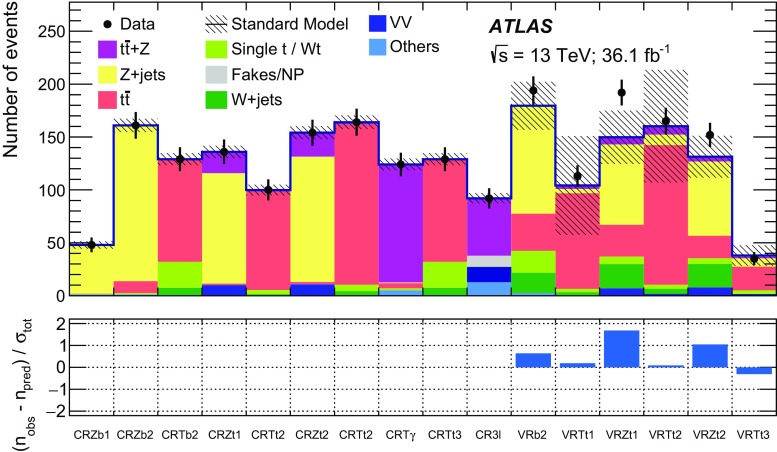
Comparison of the data with the post-fit SM prediction of the background in each control and validation region. The different background components are denoted by the colour specified in the legend. All systematic uncertainties defined in Sect. 6 and statistical uncertainties are included in the shaded band. The lower panel shows the pulls in each VR. The total uncertainty $\sigma _{\hbox {tot}}$ includes systematic and Poisson uncertainties for each given region

A second control region (CR$3\ell$), is used for the background normalisation of SRt3. It makes use of $t\bar{t} \text {+}Z$ events with $Z \rightarrow \ell ^+\ell ^-$ and semileptonic decays of the $t\bar{t}$ system (*e* or $\mu$). CR$3\ell$ is obtained by selecting three medium leptons out of which one SFOS pair is compatible with a *Z*-boson decay. This strategy allows the modelling of the lower $E_{\text {T}}^{\text {miss}}$ part of the SRt3 signal region. Additionally, the momenta of the leptons compatible with the $Z$-boson decay are added vectorially to the $\vec {p}_{\text {T}}^{\text {miss}}$ to define $\vec {p}_{\mathrm {T,\ell \ell }}^{\text {miss}}$ and $E_{\mathrm {T,\ell \ell }}^{\mathrm {miss}}$ for this control region. The transverse mass of the $\vec {p}_{\mathrm {T,\ell \ell }}^{\text {miss}}$ and the lepton not associated with the $Z$-boson decay, $m_{\mathrm {T}}^{\ell \ell }$, is combined with the $E_{\mathrm {T,\ell \ell }}^{\mathrm {miss}}$ to define a corrected $\xi ^+$: $\xi ^{+}_{\ell \ell }= m_{\mathrm {T}}^{\ell \ell }+ 0.2\cdot E_{\mathrm {T,\ell \ell }}^{\mathrm {miss}}$. A requirement is placed on this variable in CR$3\ell$ in order to approximate the kinematic properties of the signal region. The $m^{\mathrm {min}}_{b2\ell }$ variable is redefined in this region ($m^{\mathrm {min}}_{2b\ell }$) as the smaller of the two transverse masses calculated when combining the lepton not associated with the *Z*-boson decay and each of the two $b\text {-tagged}$ jets in the event.^6^ All CR selections are summarised in Table 4.

The relatively small contamination of SRt3 and CR$3\ell$ from events with fake or non-prompt (NP) leptons is estimated from data with a method similar to that described in Refs. [97, 98]. Different processes contribute to this background for the two selections. The dominant fake or non-prompt lepton contribution for SRt3 comes from semileptonic $t\bar{t}$ and $W \text {+\,jets}$ processes, while for CR$3\ell$ it comes from dileptonic $t\bar{t}$ and *Z*+*bb* processes. The method makes use of the number of observed events containing baseline–baseline, baseline–medium, medium–baseline and medium–medium lepton pairs (see definitions in Sect. 2) in a given selection. The probability for prompt leptons satisfying the baseline selection criteria to also pass the medium selection is measured using a $Z \rightarrow \ell \ell$ sample. The equivalent probability for fake or non-prompt leptons is measured from multi-jet- and $t\bar{t}$-enriched control samples. The number of events containing a contribution from one or two fake or non-prompt leptons is calculated from these probabilities.

The background from multi-jet production for the regions with no leptons is estimated from data using a procedure described in detail in Ref. [99] and modified to account for the heavy flavour of the jets. The contribution from multi-jet production in all regions is found to be very small.

Minor background contributions to each signal region are collectively called “Others” in the following. For SRb1 and SRb2, this category contains the contributions from multi-jet, single top-quark production, diboson production, $t\bar{t}$ +$V$ and $W \text {+\,jets}$. For SRt1 and SRt2, multi-jet, $V+\gamma$, diboson, single top-quark and $t\bar{t}$ production in association with Higgs or $W$ boson(s) collectively define “Others”. Finally, for SRt3 the “Others” category contains the contributions from $t\bar{t}$ +*W* / *h* / *WW*, $t\bar{t}$ $t\bar{t}$, $t\bar{t} t$, *Wh*, (*gg*)*h* and *Zh* production.

**Table 5:** Summary of the validation region selections. See Tables 2 and 3 for the detailed multi-jet rejection requirements

Observable	VRb2	VRZt1	VRZt2	VRTt1	VRTt2	VRTt3
Trigger	$E_{\text {T}}^{\text {miss}}$	$E_{\text {T}}^{\text {miss}}$		$E_{\text {T}}^{\text {miss}}$		$\scriptstyle 2\mu || 2e || 1e1\mu$
$\mathcal {N}_{j}$	$2-3$	$\ge 4$		$\ge 4$		$\ge 1$
$\mathcal {N}_{b}$	$\ge 2$	$\ge 2$		$\ge 2$		$\ge 1$
$\mathcal {N}_{\ell }$	As SR					
$\tau$ multiplicity	–	–		0		–
$E_{\text {T}}^{\text {miss}}$ [GeV]	$>180$	$>250$		$>300$		–
$p_{\text {T}} (j_1,j_2)$ [GeV]	$>150,>20$	$>80,>80$		$>80,>80$		$>30,$ –
$p_{\text {T}} (j_3,j_4)$ [GeV]	$<60,$–	$>40,>40$		$>40,>40$		–
$p_{\text {T}} (bj_1)$ [GeV]	$>150$	$>20$		$>20$		$>30$
$p_{\text {T}} (\ell _1,\ell _2)$ [GeV]	–	–		–		$>25,20$
Multi-jet rejection	As SR					
$\vert m_{\ell \ell }^{\mathrm {SF}} - m_Z \vert$ [GeV]	–	–		–		$>20$
$\delta ^-$, $\delta ^+$ [rad]	$<0, >0.5$	–		–		–
$m^{\mathrm {jet\;0}}_{\mathrm {R=SR}}$ [GeV]	–	$<80$	$<140$	$>80$	$>140$	–
$m^{\mathrm {jet\;1}}_{\mathrm {R=SR}}$ [GeV]	–	–		$>40$	$>50$	–
$m_{\mathrm {T}}^{b,\mathrm {min}}$ [GeV]	–	$>150$		(80, 150)	(100, 200)	–
$m_{\mathrm {T}}^{b,\mathrm {max}}$ [GeV]	–	$>250$	–	$>200$	–	–
$\Delta R_{bb}$	–	$<1.5$		$>0.8$	$>1.0$	–
$E_{\text {T}}^{\text {miss, sig}}$ [$\sqrt{\hbox {GeV}}$]	–	$>12$	–	–	$>10$	–
$\xi ^+$, $m^{\mathrm {min}}_{b2\ell }$, $m_{\mathrm {T2}}^{\ell \ell }$ [GeV]	–	–		–		as SR
$\Delta \phi _{\mathrm {boost}}$ [rad]	–	–		–		$>1.5$

In summary, one scaling factor is used to normalise the $Z \text {\,+\,jets}$ background in SRb1, while two scaling factors are used to normalise the $Z \text {\,+\,jets}$ and $t\bar{t}$ backgrounds in SRb2. For SRt1 and SRt2, three scaling factors for each region are used to independently normalise the $Z \text {\,+\,jets}$, $t\bar{t}$ and $t\bar{t} \text {+}Z$ backgrounds. Finally, in SRt3 the $t\bar{t}$ and $t\bar{t} \text {+}Z$ predictions are adjusted by a floating normalisation for each of the two backgrounds. The background scaling factors are treated as fully uncorrelated between the different SRs. In all selections, it is found that the normalisation of the $Z \text {\,+\,jets}$ background is larger than unity. This may be related to the fact that in the default Sherpa v2.2.1 generator the heavy-flavour production fractions are not consistent with the measured values [100]. The normalisation factors for $t\bar{t}$ processes in the SRtX regions are found to be compatible with unity, while they are found to be considerably smaller than unity for SRb2. This is due to the angular separation requirements in this region, which select $t\bar{t}$ events in a specific corner of the phase space. Finally, the different normalisations of the $t\bar{t} \text {+}Z$ background processes found in the CR$\gamma$ and CR$3\ell$ regions (larger and smaller than unity, respectively) are due to the different kinematic requirements on the jet momenta and the corrected $E_{\text {T}}^{\text {miss}}$ in the two regions, which are designed to mimic the topology of the respective signal regions.

Dedicated validation regions are used to validate the background prediction for each of the SRs and evaluate the reliability of the MC extrapolation of the SM background estimates from CRs to SRs. The background estimates in SRb2 are validated in a single VR (VRb2) which has a background composition similar to that of the SR. Selected key distributions in the control and validation regions are shown in Fig. 2. The prediction of the $Z \text {\,+\,jets}$ background in SRb1 relies on an extrapolation over a large interval of missing transverse momentum. As CRZb1 is designed to be kinematically as close as possible to SRb1 and given the low yield in this region, it was not possible to construct a selection to validate this extrapolation. Nevertheless, the use of the same kinematic selection in control and signal region, together with the good agreement between the data and the post-fit SM prediction in CRZb1 in the whole $E_{\mathrm {T,\ell \ell }}^{\mathrm {miss}}$ spectrum (Fig. 2) gives confidence in the accuracy of the estimate. Two validation regions, VRZt1 and VRZt2, are designed to validate the $Z \text {\,+\,jets}$ estimate in SRt1 and SRt2. Furthermore, the top background estimate in these SRs is validated in two additional VRs: VRTt1 and VRTt2. Finally, VRTt3 is designed to validate the top background prediction in SRt3. All requirements for each validation region are summarised in Table 5. The data and the post-fit Monte Carlo background prediction yields in each CR and VR are compared in Fig. 3. The background yields in the control regions match the observed data by construction. In the validation regions, the background prediction is compatible with the observed data within two standard deviations of the total systematic uncertainty.

## Systematic uncertainties

Experimental and theoretical sources of systematic uncertainty in the signal and background estimates are considered in this analysis. Their impact is constrained overall through the normalisation of the dominant backgrounds in the control regions defined with kinematic selections resembling those of the corresponding signal region.

The dominant sources of detector-related systematic uncertainty are the jet energy scale, the jet energy resolution, the *b*-tagging efficiency and mis-tagging rates, and the scale and resolution of the $E_{\text {T}}^{\text {miss}}$ soft term. The jet energy scale and resolution uncertainties are derived as a function of the $p_{\text {T}}$ and $\eta$ of the jet, as well as of the pile-up conditions and the jet flavour composition of the selected jet sample [37]. Uncertainties associated with the modelling of the *b*-tagging efficiencies for *b*-jets, *c*-jets and light-flavour jets [101, 102] are derived as a function of $\eta$, $p_{\text {T}}$ and flavour of each jet. The systematic uncertainties related to the modelling of $E_{\text {T}}^{\text {miss}}$ in the simulation are estimated by propagating the uncertainties in the energy and momentum scale of all identified electrons, photons, muons and jets, as well as the uncertainties in the soft-term scale and resolution [49]. Other detector-related systematic uncertainties, such as those in the lepton and photon reconstruction efficiency, energy scale and energy resolution, and in the modelling of the trigger [43], are found to have a small impact on the results.

**Table 6:** Summary of the main systematic uncertainties and their impact on the total SM background prediction in each of the signal regions studied. A range is shown for the four bins composing SRb2. The total systematic uncertainty can be different from the sum in quadrature of individual uncertainties due to the correlations between them resulting from the fit to the data

	SRb1 (%)	SRb2 (%)	SRt1 (%)	SRt2 (%)	SRt3 (%)
Total systematic uncertainty	18	15–18	29	14	28
$Z$ theoretical uncertainties	5.7	7.9–12	5.0	2.1	$<1$
$t\bar{t} \text {+}Z$ theoretical uncertainties	$<1$	$<1$	3.3	5.3	8.4
$t\bar{t}$ theoretical uncertainties	$<1$	2.7–9.8	17	5.7	11
MC statistical uncertainties	6.4	4.8–6.4	15	5.9	18
$Z$ fitted normalisation	13	12–19	2.3	3.4	–
$t\bar{t} \text {+}Z$ fitted normalisation	–	–	2.2	3.5	7.1
$t\bar{t}$ fitted normalisation	–	1.9–4.2	3.9	1.4	2.0
Fake or non-prompt leptons	–	–	–	–	7.9
Pile-up	3.8	$<1-1.4$	6.8	5.5	$<1$
Jet energy resolution	1.5	1.3–6.9	7.0	$<1$	$<1$
Jet energy scale	7.7	5.0–10	5.0	2.8	8.2
$E_{\text {T}}^{\text {miss}}$ soft term	$<1$	4.3–6.3	2.0	$<1$	12
*b*-tagging	$<1$	2.4–6.9	8.6	3.1	$<1$

Uncertainties in the theoretical modelling of the SM background processes from MC simulation are also taken into account. The uncertainties in the modelling of the $t\bar{t}$ process are estimated by varying the renormalisation and factorisation scales, as well as the amount of initial- and final-state radiation used to generate the samples  [55]. The uncertainty connected with the parton-shower modelling is estimated as the difference between the predictions from Powheg showered with Pythia or Herwig. Additionally, the uncertainty related to the choice of event generator is evaluated by comparing the Powheg and MadGraph5_aMC@NLO predictions [55] for SRb1, SRb2 and SRt3. Due to the higher jet multiplicity required in SRt1 and SRt2 the generator uncertainty is evaluated instead by comparing the Powheg and Sherpa predictions. The uncertainties in the modelling of the $Z$ background are accounted for by varying the default renormalisation, factorisation, resummation and matching scales of the Sherpa samples. For SRt1 and SRt2 an additional uncertainty is included to account for effects on the $\Delta R_{bb}$ modelling not captured by the scale variations. This is estimated as the difference between the observed yield in data and the post-fit background prediction plus one times its uncertainty in each of the VRZs. The theoretical uncertainty connected with the $t\bar{t} Z$ background in SRt1 and SRt2 is estimated by varying independently the renormalisation, factorisation, resummation and matching scales in the $t\bar{t} Z$ and $t\bar{t} \gamma$ samples in signal and control regions, respectively. PDF uncertainties (estimated by varying the parametrisation of the PDF set used to generate the simulated background samples) are found to have a non-negligible impact for this background component and are treated as correlated between signal and control regions. An additional uncertainty in the extrapolation between control and signal region is derived as the difference between the ratio of the $t\bar{t} \gamma$ and $t\bar{t} Z$ cross-section predictions obtained with the nominal MC generator and with the alternative MC generator Sherpa interfaced to OpenLoops. For SRt3, SRb1 and SRb2 the uncertainty connected with the $t\bar{t} Z$ background estimation is assessed by varying the renormalisation, factorisation, resummation and matching scales.

Systematic uncertainties are assigned to the estimated background from fake or non-prompt leptons in SRt3 to account for potentially different compositions (heavy flavour, light flavour or conversions) between the signal regions and the control regions used for the fake-rate extraction, as well as the contamination from prompt leptons in the regions used to measure the probabilities for loose fake or non-prompt leptons to satisfy the tight signal criteria. Table 6 summarises the contributions from the different sources of systematic uncertainty in the total SM background predictions for the different signal regions after the fit to the control regions described in Sect. 5. As can be seen, the contribution from the theoretical uncertainty in the $t\bar{t}$ background and the contribution from the statistical uncertainty connected with the use of Monte Carlo simulations are higher in SRt1 than in SRt2. The reason for the higher contribution from the theoretical uncertainty in the $t\bar{t}$ background is primarily due to the larger relative importance of this source of background in SRt1. The reason for the higher contribution from the statistical uncertainty is connected with the *W*-boson background, which is predicted with low statistical precision in SRt1.

The impact of theoretical and detector-related uncertainties on the dark-matter signal acceptance is considered. The same procedure used to evaluate background uncertainties is applied for the detector-related uncertainties. The theoretical uncertainties in the acceptance are assessed by varying the factorisation, renormalisation, matching scales and parton shower parameters. For SRb1 the total theoretical uncertainty in the acceptance is 6%, for SRb2 it is below 8%, and for SRt1, SRt2 and SRt3 it ranges from 10 to 12%. The theoretical uncertainties in the production cross-section of the signal are evaluated only for the colour-neutral mediator models, for which an NLO computation of the cross-section is available. It is estimated by considering the same scale variations used to assess the uncertainties in the acceptance, and by varying the parametrisation of the PDF set used to generate the simulated signal samples. An additional uncertainty due to the different scale adopted to evaluate the NLO cross-section and to generate the signal samples is also considered. The total theoretical uncertainty in the cross-section amounts to 9% for the on-shell regime in the mass range of $t\bar{t} +\phi /a$ signals to which the analysis is sensitive, and ranges from 9 to 30% for the off-shell regime. For the $b\bar{b} +\phi /a$ signals this uncertainty varies between 5 and 13%.

## Results

**Table 7:** Fit results in SRb1 and SRb2 for an integrated luminosity of $36.1 \; \mathrm {fb}^{-1}$. The background normalisation parameters are obtained from the background-only fit in the CRs and are applied to the SRs. Pre-fit values are also shown. Small backgrounds are indicated as Others (see text for details). The dominant component of these smaller background sources in SRb1 is diboson processes. Benchmark signal models yields are given for each SR. The uncertainties in the yields include statistical uncertainties and all systematic uncertainties defined in Sect. 6

	SRb1	SRb2-bin1	SRb2-bin2	SRb2-bin3	SRb2-bin4
Observed	19	88	88	90	82
Total background (fit)	$16.9 \pm 3.3$	$77 \pm 13$	$72 \pm 11$	$76 \pm 13$	$66.4 \pm 9.1$
$Z/\gamma ^* \text {+ jets}$	$14.2 \pm 3.1$	$39.7 \pm 6.3$	$44.4 \pm 6.6$	$53.3 \pm 9.9$	$55.6 \pm 8.6$
$t\bar{t}$	$0.58_{-0.58}^{+0.60}$	$17.8 \pm 6.5$	$13.8 \pm 5.5$	$14.0 \pm 4.7$	$7.0 \pm 2.9$
Single top quark	$0.25_{-0.25}^{+0.42}$	$14.7 \pm 5.8$	$10.2 \pm 3.7$	$5.5 \pm 3.1$	$2.6 \pm 1.7$
Others	$2.0 \pm 1.1$	$5.2 \pm 3.4$	$3.4_{-1.6}^{+1.7}$	$2.7 \pm 1.1$	$1.3 \pm 1.0$
$Z/\gamma ^* \text {+ jets}$ (pre-fit)	12.1	30.6	34.2	41.1	42.8
$t\bar{t}$ (pre-fit)	–	27.1	21.1	21.4	10.6
Signal benchmarks					
$m(\phi ,\chi )=(20,1)\;\hbox {GeV}$, $g=1$		$0.238\pm 0.085$	$0.262\pm 0.079$	$0.320\pm 0.082$	$0.277\pm 0.080$
$m(a,\chi )=(20,1)\;\hbox {GeV}$, $g=1$		$0.256\pm 0.065$	$0.199\pm 0.060$	$0.308\pm 0.085$	$0.267\pm 0.067$
$m(\phi _b,\chi )=(1000,35)\;\hbox {GeV}$	$18.6\pm 3.8$

**Table 8:** Fit results in SRt1, SRt2 and SRt3 for an integrated luminosity of $36.1 \; \mathrm {fb}^{-1}$. The background normalisation parameters are obtained from the background-only fit in the CRs and are applied to the SRs. Pre-fit values are also shown. Small backgrounds are indicated as Others (see text for details). Benchmark signal models yields are given for each SR. The uncertainties in the yields include statistical uncertainties and all systematic uncertainties defined in Sect. 6

	SRt1	SRt2	SRt3
Observed	23	24	18
Total background (fit)	$20.5\pm 5.8$	$20.4\pm 2.9$	$15.2 \pm 4.3$
$t\bar{t}$	$7.0\pm 3.9$	$3.1\pm 1.3$	$4.5 \pm 2.5$
$t\bar{t}$+*Z*	$4.3\pm 1.1$	$6.9\pm 1.4$	$4.4 \pm 1.9$
$W \text {+\,jets}$	$3.3\pm 2.6$	$1.28\pm 0.50$	Incl. in fakes/NP
*Wt*	Incl. in others	Incl. in others	$0.33_{-0.33}^{+0.53}$
$Z/\gamma ^* \text {+ jets}$	$3.7\pm 1.4$	$6.2\pm 1.1$	Incl. in others
*VV*	Incl. in others	Incl. in others	$0.61 \pm 0.25$
Fakes/NP	–	–	$2.7 \pm 1.3$
Others	$2.2\pm 1.2$	$3.00\pm 1.6$	$2.69 \pm 0.93$
$t\bar{t}$ (pre-fit)	6.1	2.8	4.0
$t\bar{t}$+*Z* (pre-fit)	3.53	5.6	5.6
$Z/\gamma ^* \text {+ jets}$ (pre-fit)	3.2	5.72	–
Signal benchmarks			
$m(\phi ,\chi )=(20,1)\;\hbox {GeV}$, $g=1$	$9.3 \pm 1.6$	$12.8 \pm 1.9$	$21.0 \pm 2.3$
$m(a,\chi )=(20,1)\;\hbox {GeV}$, $g=1$	$7.6 \pm 1.5$	$12.1 \pm 1.8$	$14.1 \pm 1.6$
$m(\phi ,\chi )=(100,1)\;\hbox {GeV}$, $g=1$	$6.5 \pm 1.3$	$10.1 \pm 1.5$	$11.5 \pm 1.5$
$m(a,\chi )=(100,1)\;\hbox {GeV}$, $g=1$	$6.2 \pm 1.2$	$11.5 \pm 2.0$	$11.9 \pm 1.5$

**Table 9:** Left to right: 95% CL upper limits on the visible cross-section ($\langle \epsilon \mathcal {A}\sigma \rangle ^\mathrm{obs}_{95}$) and on the number of BSM events ($S^\mathrm{obs}_{95}$). The third column ($S^\mathrm{exp}_{95}$) shows the 95% CL upper limit on the number of signal events, given the expected number (and $\pm 1\sigma$ excursions of the expected number) of background events. The last column indicates the discovery *p*-value ($p(s = 0)$) and *Z* (the number of equivalent Gaussian standard deviations)

Signal channel	$\langle \epsilon \mathcal {A}\mathrm{\sigma }\rangle ^\mathrm{obs}_{95}$ [fb]	$S^\mathrm{obs}_{95}$	$S^\mathrm{exp}_{95}$	$p(s=0)$ (*Z*)
SRb1	0.37	13.4	$$ { 12 }^{ +5 }_{ -1 }$$	0.33 (0.43)
SRb2 bin-1	1.10	39.6	$$ { 33 }^{ +12 }_{ -8 }$$	0.22 (0.76)
SRb2 bin-2	1.17	42.1	$$ { 31 }^{ +10 }_{ -8 }$$	0.11 (1.21)
SRb2 bin-3	1.21	43.7	$$ { 33 }^{ +11 }_{ -8 }$$	0.16 (1.00)
SRb2 bin-4	1.10	39.8	$$ { 26 }^{ +11 }_{ -7 }$$	0.10 (1.26)
SRt1	0.51	18.4	$$ { 16 }^{ +5 }_{ -4 }$$	0.33 (0.44)
SRt2	0.44	15.7	$$ { 12 }^{ +5 }_{ -3 }$$	0.24 (0.70)
SRt3	0.44	15.9	$$ { 13 }^{ +5 }_{ -2 }$$	0.33 (0.45)

The expected and observed yields in each of the five signal regions of this analysis are reported in Tables 7 and 8. The background-only fit to the control regions described in Sect. 5 is compared to the predictions based on the MC normalisation. The observed data is found to be compatible with the background prediction in each one of the SRs. The expected signal yields for selected benchmark models for colour-neutral and colour-charged mediators are also shown. In each SR the observed yield in data is above the expected background but within 1.3 standard deviations of its uncertainty.

**Fig. 4:**
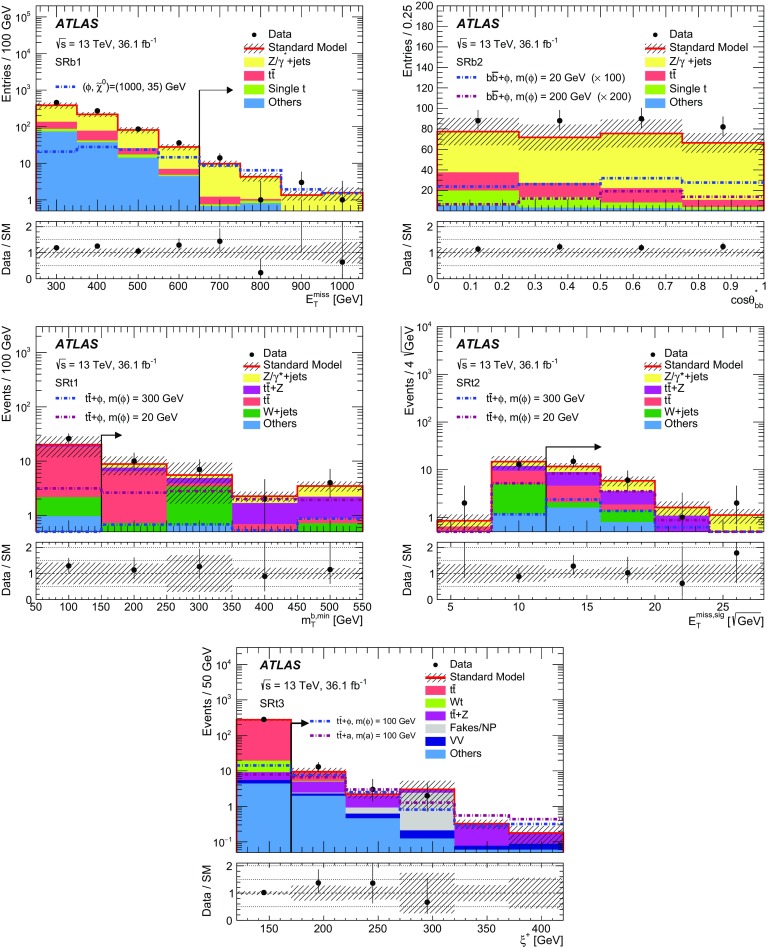
Comparison of the data with the post-fit SM prediction of the $E_{\text {T}}^{\text {miss}}$ distribution in SRb1 (top left), $\cos {\theta }^*_{bb}$ distribution in SRb2 (top right), $m_{\mathrm {T}}^{b,\mathrm {min}}$ distribution in SRt1 (middle left), $E_{\text {T}}^{\text {miss, sig}}$ distribution in SRt2 (middle right) and $\xi ^+$ distribution in SRt3 (bottom). The last bins include overflows, where applicable. All signal region requirements except the one on the distribution shown are applied. The signal region requirement on the distribution shown is indicated by an arrow. The bottom panel shows the ratio of the data to the prediction. The band includes all systematic uncertainties defined in Sect. 6

Figure 4 shows a comparison between the SM predictions and the observed data for some relevant kinematic distributions in each signal region prior to the selection on the variable. The four bins of SRb2 are statistically combined in the final result. A model-independent fit set-up [96] where both the control and signal regions are included in the fit is used to derive 95% confidence level (CL) upper limits on the visible cross-section $\langle \epsilon \mathcal {A}\mathrm{\sigma }\rangle _{95}$ of new physics beyond-the-SM (BSM) processes, defined as cross-section times acceptance times efficiency and obtained as the upper limit on the number of BSM events divided by the total integrated luminosity. The 95% CL exclusion limits are derived with the CL$_\mathrm {s}$ method [103] and summarised in Table 9 for each SR. These limits are calculated assuming no systematic uncertainties for the signal and neglecting any possible signal contamination in the control regions.

The results are also used to set limits on the production cross-section of colour-neutral and colour-charged mediator models decaying into dark-matter particles. An independent fit is used for each of the five signal regions. When deriving model-dependent limits, the expected signal yield in each fit region is considered.

For the signal, the experimental systematic uncertainties and theoretical systematic uncertainties in the acceptance are taken into account for this calculation. The experimental uncertainties are assumed to be fully correlated with those in the SM background. The theoretical systematic uncertainties in the signal cross-section are instead shown separately in the final exclusion result for the colour-neutral mediator models.

**Fig. 5:**
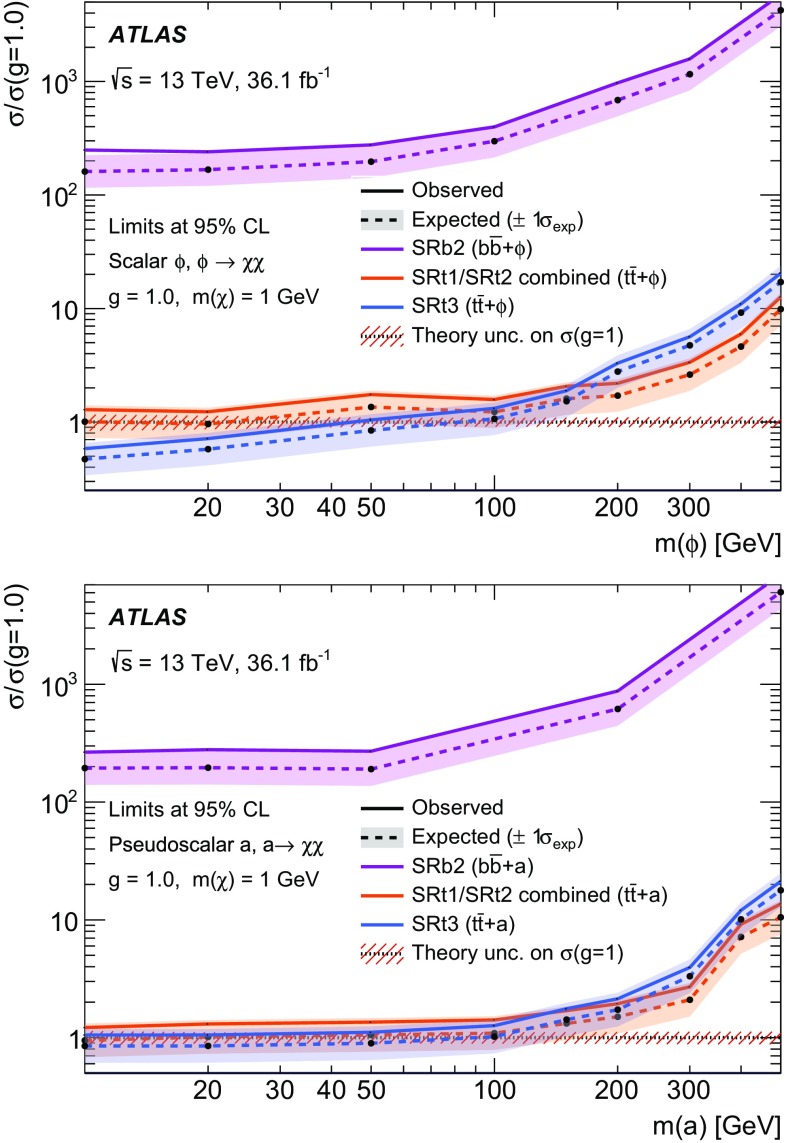
Exclusion limits for colour-neutral $t\bar{t}/b\bar{b} +\phi$ scalar (top) and $t\bar{t}/b\bar{b} +a$ pseudoscalar (bottom) models as a function of the mediator mass for a DM mass of $1\;\hbox {GeV}$. The limits are calculated at 95% CL and are expressed in terms of the ratio of the excluded cross-section to the nominal cross-section for a coupling assumption of $g = g_q = g_\chi = 1$. The solid (dashed) lines shows the observed (expected) exclusion limits for the different signal regions, according to the colour code specified in the legend. To derive the results for the fully hadronic $t\bar{t}$ final state the region SRt1 or SRt2 providing the better expected sensitivity is used

**Fig. 6:**
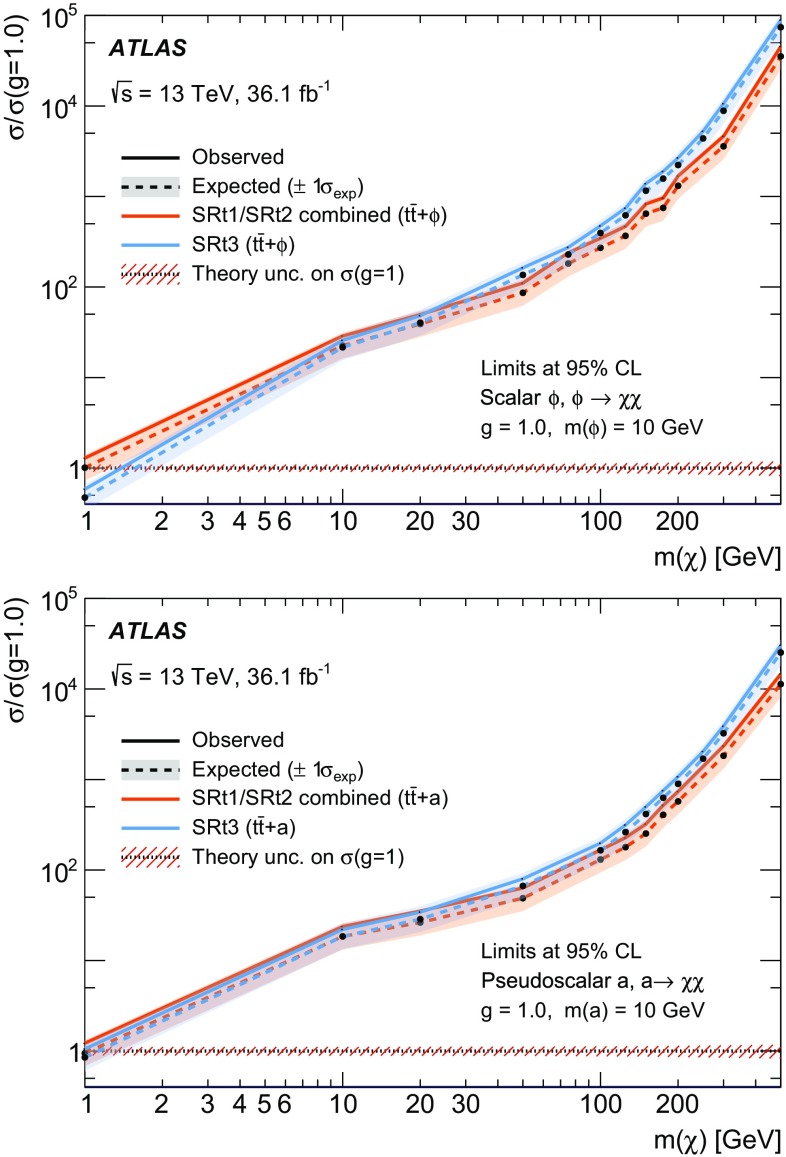
Exclusion limits for colour-neutral $t\bar{t} +\phi$ scalar (top) and $t\bar{t} +a$ pseudoscalar (bottom) models as a function of the DM mass for a mediator mass of $10\;\hbox {GeV}$. The limits are calculated at 95% CL and are expressed in terms of the ratio of the excluded cross-section to the nominal cross-section for a coupling assumption of $g = g_q = g_\chi = 1$. The solid (dashed) lines shows the observed (expected) exclusion limits for the different signal regions, according to the colour code specified in the legend. To derive the results for the fully hadronic $t\bar{t}$ final state the region SRt1 or SRt2 providing the better expected sensitivity is used

Figures 5 and 6 show upper limits at 95% CL on the signal cross-section scaled to the signal cross-section for coupling $g=1$, denoted by $\sigma /\sigma (g=1.0)$. These are the most stringent limits to date on $t\bar{t} +\phi /a$ models and the first ATLAS results for the $b\bar{b} +\phi /a$ models. To derive the results for the fully hadronic $t\bar{t}$ final state the region SRt1 or SRt2 providing the better expected sensitivity is used. The SRt1 was originally optimised for low-mass scalar mediators, while SRt2 was optimised for high-mass scalar mediators and pseudoscalar mediators. However, SRt1 is strongly affected by systematic uncertainties in the $t\bar{t}$ modelling and therefore SRt2 sets more stringent limits for the whole parameter space. These limits are obtained both as a function of the mediator mass, assuming a specific DM mass of $1\; \hbox {GeV}$ (Fig. 5), and as a function of the DM mass, assuming a specific mediator mass of $10\;\hbox {GeV}$ (Fig. 6). Both the scalar and pseudoscalar mediator cases are considered. The sensitivity for $t\bar{t} +\phi /a$ on-shell decays is approximately constant for masses below $100\;\hbox {GeV}$, with SRt3 excluding the $g=1$ assumption for scalar mediator masses up to $50\;\hbox {GeV}$. For a given mediator mass the acceptance of the analysis is independent of the value of the DM mass as long as $m(\phi /a) > 2\cdot m(\chi )$ is fulfilled and width effects can be neglected. Under these conditions, exclusion limits for DM masses differing from the one presented can be inferred from the result shown in Fig. 5. Due to the smaller Yukawa enhancement of $b\bar{b} +\phi /a$ final states, it is possible to exclude cross-sections 300 times the nominal values for $g=1$.

**Fig. 7:**
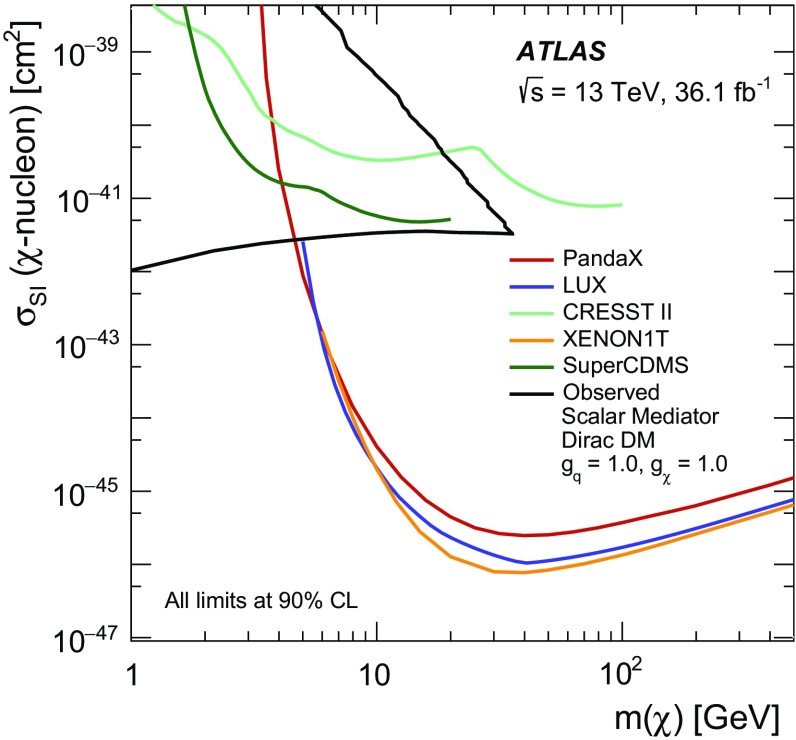
Comparison of the 90% CL limits on the spin-independent DM–nucleon cross-section as a function of DM mass between these results and the direct-detection experiments, in the context of the colour-neutral simplified model with scalar mediator. The black line indicates the exclusion contour derived from the observed limits of SRt3. Values inside the contour are excluded. The exclusion limit is compared with limits from the LUX [104], PandaX-II [105], XENON [106], SuperCDMS [107] and CRESST-II [108] experiments

**Fig. 8:**
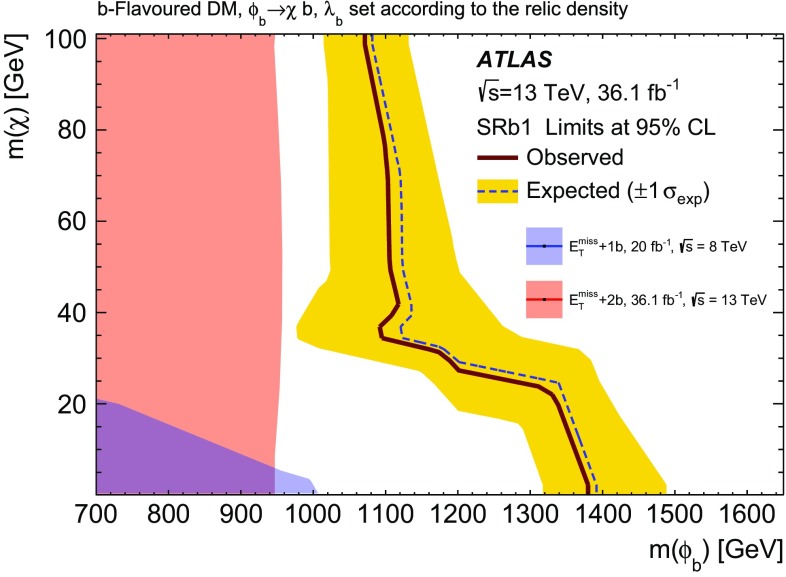
Exclusion limits for colour-charged scalar mediators ($b$-FDM) as a function of the mediator and DM masses for $36.1 \; \mathrm {fb}^{-1}$ of data. The limits are calculated at 95% CL. The solid (dashed) line show the observed (expected) exclusion contour for a coupling assumption $\lambda _b$ yielding the measured relic density. No uncertainties on the LO cross-sections are considered for this model. The results are compared with the ATLAS search for $b$-FDM models [27], represented by the blue contour, and the ATLAS search for direct sbottom pair production [25], represented by the red contour

For each dark-matter and mediator mass pair, the exclusion limit on the production cross-section of colour-neutral scalar mediator particles can be converted into a limit on the spin-independent DM–nucleon scattering cross-section using the procedure described in Ref. [109]. The results can thus be compared with the results from direct-detection experiments. The most stringent limits, provided by SRt3, are used for this purpose. Figure 7 shows the constraints from this analysis expressed as exclusion limits at 90% CL in the plane defined by the dark-matter mass and the scattering cross-section. The black line indicates the exclusion contour derived from the observed limits in the top part of Fig. 5, where mediator masses between 10 GeV and 500 GeV are considered. The maximum value of the DM–nucleon scattering cross-section displayed corresponds to the result obtained for a mediator mass of 10 GeV. The results of this analysis are compared with the results from the LUX [104], PandaX-II [105], XENON [106], SuperCDMS [107] and CRESST-II [108] experiments. The comparison is model-dependent, and therefore valid only for the specific models considered in this paper. For pseudoscalar mediator models, the predicted dark-matter cross-sections in these direct-detection experiments is suppressed by velocity-dependent terms. As a result, direct-detection limits on spin-independent DM–nucleon scattering cross-section are several orders of magnitude worse than the ones obtained in this analysis, and therefore not presented.

Finally, Fig. 8 shows the exclusion contour for the $b$-FDM model as a function of the mediator and DM masses. In this model, the cross-section and therefore also the final sensitivity strongly depends on the coupling choice, $\lambda _b$, which is set to fulfil the relic density constraints, and determines the decrease of the sensitivity for higher DM masses. For a DM particle of approximately $35\; \hbox {GeV}$, as suggested by the interpretation of data recorded by the Fermi-LAT Collaboration, mediator masses below $1.1\; \hbox {TeV}$ are excluded at 95% CL.

## Conclusion

This article reports a search for dark-matter pair production in association with bottom or top quarks. The analysis is performed using $36.1 \; \mathrm {fb}^{-1}$ of *pp* collisions collected at a centre-of-mass energy of $\sqrt{s} = 13~\hbox {TeV}$ by the ATLAS detector at the LHC. The results are interpreted in the framework of simplified models of spin-0 mediators to the dark sector decaying into pairs of DM particles. The data are found to be consistent with the Standard Model expectations, and limits are set on the signal strength for a coupling assumption of $g=1.0$ or on the DM and mediator masses. The results represent the most stringent limits to date for colour-neutral spin-0 mediator models for a DM mass assumption of $1\;\hbox {GeV}$ in top-quark final states. It excludes at 95% CL mediator masses between 10 and $50\; \hbox {GeV}$ for scalar mediators assuming couplings equal to unity and a dark-matter mass of $1\;\hbox {GeV}$. Although the analysis is expected to be sensitive to models with pseudoscalar mediators with masses between 10 and 100 GeV, no observed exclusion limit can be set for this model for the coupling assumption of $g=1.0$ because of a small excess in the observed data. Limits of 300 times the nominal cross section for couplings equal to unity are placed for scalar and pseudoscalar mediator masses between 10 and $50\; \hbox {GeV}$ for a dark-matter mass of $1\;\hbox {GeV}$ in bottom-quark final states. Constraints on $b$-FDM models are also presented. The excluded region depends on $m(\phi _b)$ and $m(\chi )$; for $m(\chi ) = 35\; \hbox {GeV}$, mediator particles with $m(\phi ) < 1.1\; \hbox {TeV}$ are excluded.

## Footnotes

1 Following Ref. [14], couplings to *W* and *Z* bosons, as well as explicit dimension-4 $\phi$–*h* or *a*–*h* couplings, are set to zero in this simplified model. In addition, the coupling of the mediator to the dark sector are not taken to be proportional to the mass of the DM candidates.

2 ATLAS uses a right-handed coordinate system with its origin at the nominal interaction point (IP) in the centre of the detector and the *z*-axis along the beam pipe. The *x*-axis points from the IP to the centre of the LHC ring, and the *y*-axis points upward. Cylindrical coordinates (*r*, $\phi$) are used in the transverse plane, $\phi$ being the azimuthal angle around the beam pipe. The pseudorapidity is defined in terms of the polar angle $\theta$ as $\eta = -\ln \tan (\theta /2)$. Rapidity is defined as $y=0.5 \ln \left[ (E + p_z )/(E - p_z )\right]$ where *E* denotes the energy and $p_z$ is the component of the momentum along the beam direction.

3 The transverse mass of two particles *a* and *b* is defined as $m_{\mathrm T}(a,b) = \sqrt{(E_{\mathrm T,a}+E_{\mathrm T,b})^2 - (\vec {p}_{\mathrm T,a}+\vec {p}_{\mathrm T,b})^2}$

4 The angular separation between two particles *a*, *b* used in all quantities described in this section is defined as $\Delta R_{ab} = \sqrt{(\Delta \phi _{ab})^2+(\Delta \eta _{ab})^2}$.

5 The transverse mass in this case is calculated by neglecting the lepton masses.

6 When the $b\text {-tagged}$ jet multiplicity is different from two, the two jets with the highest *b*-tagging probabilities are chosen, independently of whether they are $b\text {-tagged}$ or not.
